# MitoRCA-seq reveals unbalanced cytocine to thymine transition in *Polg* mutant mice

**DOI:** 10.1038/srep12049

**Published:** 2015-07-27

**Authors:** Ting Ni, Gang Wei, Ting Shen, Miao Han, Yaru Lian, Haihui Fu, Yan Luo, Yanqin Yang, Jie Liu, Yoshi Wakabayashi, Zheng Li, Toren Finkel, Hong Xu, Jun Zhu

**Affiliations:** 1 State Key Laboratory of Genetic Engineering & Ministry of Education (MOE) Key Laboratory of Contemporary Anthropology, Collaborative Innovation Center of Genetics and Development, School of Life Sciences, Fudan University, Shanghai, 200438, P.R. China; 2 Genetics and Development Biology Center, National Heart Lung Blood Institute, National Institutes of Health, Bethesda, Maryland 20892, USA; 3 Laboratory of Molecular Biology, National Heart Lung Blood Institute, National Institutes of Health, Bethesda, Maryland 20892, USA; 4 Unit on Synapse Development and Plasticity, National Institute of Mental Health, National Institutes of Health, Bethesda, Maryland 20892, USA

## Abstract

Mutations in mitochondrial DNA (mtDNA) can lead to a wide range of human diseases. We have developed a deep sequencing strategy, mitoRCA-seq, to detect low-frequency mtDNA point mutations starting with as little as 1 ng of total DNA. It employs rolling circle amplification, which enriches the full-length circular mtDNA by either custom mtDNA-specific primers or a commercial kit, and minimizes the contamination of nuclear encoded mitochondrial DNA (Numts). By analyzing the mutation profiles of wild-type and *Polg* (mitochondrial DNA polymerase γ) mutant mice, we found that mice with the proofreading deficient mtDNA polymerase have a significantly higher mutation load by expanding the number of mutation sites and to a lesser extent by elevating the mutation frequency at existing sites even before the premature aging phenotypes appear. Strikingly, cytocine (C) to thymine (T) transitions are found to be overrepresented in the mtDNA of *Polg* mutated mice. The C → T transition, compared to other types of mutations, tends to increase the hydrophobicity of the underlying amino acids, and may contribute to the impaired protein function of the *Polg* mutant mice. Taken together, our findings may provide clues to further investigate the molecular mechanism underlying premature aging phenotype in *Polg* mutant mice.

Mitochondrial DNA (mtDNA) encodes important genetic information in addition to the nuclear genome. Unlike the diploid nuclear genome, each mammalian cell contains thousands of copies of mtDNAs. The heteroplasmic nature of mtDNA mutations make their analysis more challenging^1^,^2^. Mutations in mtDNAs, especially the high-frequency point mutations, have been linked to more than 100 human disorders^3^,^4^, including cardiovascular disease, diabetes, deafness and Parkinson’s disease. Emerging studies have begun to characterize the significance of low-frequency mtDNA mutations in tumor progression and aging^2^,^3^,^5^,^6^,^7^. Moreover, recent studies indicated that low-level mtDNA heteroplasmy can affect the progeny’s phenotypes through unbalanced transmission between generations^7^,^8^. Thus, there is a need for a reliable method to detect low-abundance point mutations in the mitochondrial genome.

Recent advances in high-throughput sequencing technology had led to several strategies for global identification of mtDNA mutations^9^. In these methods, mitochondrial DNA fragments are enriched by direct isolation of mitochondrion^1^, multiplex Polymerase Chain Reaction (PCR)^2^,^10^,^11^,^12^ or capture-based approach with mtDNA-specific oligonucleotides^2^,^11^,^13^,^14^. However, each approach has its own limitations. Isolation of mitochondria is labor-intensive and not practical for large number of samples or circumstances with very limited material, for which mitochondria cannot be isolated (e.g. paraffin-embedded tissues). One major challenge for PCR and oligonucleotide-capture based methods is the contamination of Numts, the DNA sequences in the nucleus with high homology to mtDNA^15^,^16^. There are hundreds of Numts in the mouse and human genomes, overlapping with 84.5% and 99.99% of the mtDNA sequences in the respective organism (see Supplementary Methods for details). The presence of Numts obscures the identification of real point mutations in mtDNA especially those occurring at a low frequency^11^,^14^,^17^.

The mice expressing a proofreading-deficient version of the mitochondrial DNA polymerase γ (POLG) provide an excellent model to study low-frequency mtDNA mutations and the related phenotypes (e.g., decreased body weight, kyphosis, reduced hair density and induction of apoptosis)^5^,^18^,^19^,^20^. By mutating a critical aspartate residue in the POLG exonuclease domain to alanine (D257A), the mtDNA polymerase γ showed reduced proofreading activity during mtDNA replication^5^,^20^. Sanger sequencing results showed that mice containing the homozygous *Polg* mutated allele (denote *Polg* mutant hereafter) have a higher load (~3–5 times) of somatic mtDNA point mutations for *Cytb* (Cytochorme b) gene and the control region (or D-loop region) in brain, liver and heart^5^. To obtain a full spectrum of somatic mutations for mitochondrial genome, Ameur *et al.* sequenced the whole mtDNA region from *Polg* mutant mice by SOLiD platform^1^. They found homozygous *Polg* mutant mice have a median point mutation load of 12 × 10^−4^ per site, while the wild-type mice have mutational load of 1.3–1.8 × 10^−4^. Mutational load can be separated into the number of mutation sites and the frequency at individual sites. When 0.5% was used as the minimum mutation frequency, Ameur *et al.* found homozygous *Polg* mutant mice have more mutation sites (~5.3–6.6 times) compared with wild-type mice at 30–40 weeks of age^1^. Although Trifunovic *et al.* had examined 2-month old *Polg* mutant and wild-type mice for mutations in the *Cytb* gene and control region^5^, it remains unclear whether there is a global change in mitochondrial somatic mutation spectrum before the premature ageing phenotypes becoming apparent at ~25 weeks. If so, how this might contribute to the development of premature ageing phenotypes at the molecular level?

In this study, we developed mitoRCA-seq, a simple and robust mitochondrial DNA sequencing procedure, to compare the mutational spectrum of 6-week old wild-type and *Polg* mutant mice (early stage before premature phenotypes appear). MitoRCA-seq employs rolling circle amplification (RCA), which has two advantages: (i) It amplifies full-length mitochondrial DNA and thus minimize Numt contamination; (ii) mtDNA can be enriched from as little as 1 ng of total DNA, which makes it suitable for broad applications. The reliability of mitoRCA-seq is demonstrated by profiling (i) different amount of total DNA (1 ng, 5 ng and 50 ng) or (ii) technical replicates starting from different RCA reactions. Strikingly, we found that mutational load is increased in the *Polg* mutant mice, by expanding the number of mutation sites and elevating mutation frequency at the existing sites, at the age of 6 weeks. The time point is much earlier than the 25-week old when the premature ageing phenotypes start to appear^5^. Further analyses revealed that cytocine (C) to thymine (T) transitions are overrepresented among all point mutations, and specific to *Polg* mutant mice in a context dependent manner. We further demonstrated that C → T transitions often lead to an increase in the hydrophobicity of the underlying amino acids. These findings suggest that mutational events are ahead of the cellular changes and accumulation of mtDNA mutations might ultimately contribute to premature ageing phenotype.

## Results

### Development of a simplified mtDNA deep sequencing method

**Figure 1:**
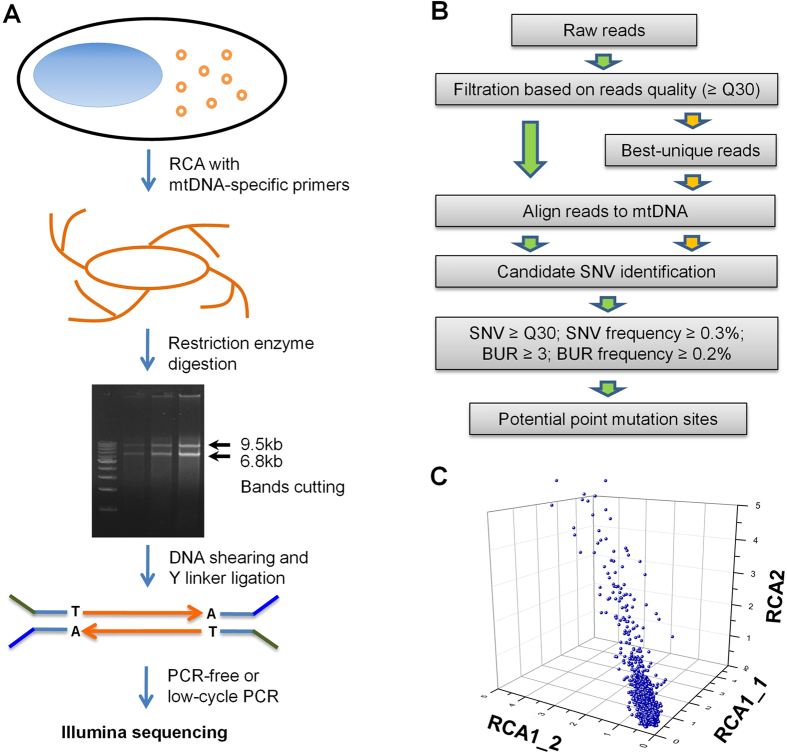
An overview of the mitoRCA-seq procedure. (**A**) Mitochondrial DNA is enriched by RCA using mtDNA specific primers in a single tube. Further enrichment of mtDNA fragments is achieved by restriction enzyme digestion (EcoRV for mouse), and only full-length mouse mtDNA can give rise to two discrete DNA fragments (mouse: 9.5 kb and 6.8 kb). Different starting materials (1 ng, 5 ng and 50 ng total DNA, from left to right) were tested. (**B**) The computational workflow employed to identify mtDNA mutations. For raw reads with the same sequence, only one read was kept for downstream mutation calling, which has the highest average quality score (defined as best-unique read). (**C**) The overall correlation of three technical replicates derived from the liver of a *Polg* mutant mouse. The frequencies of individual mutations identified in each library are plotted. RCA1-1 and RCA1-2 represents two libraries prepared from the same RCA reaction. RCA2 is a technical replicate prepared from a different RCA reaction. The correlation coefficient (R) between technical replicates is 0.998 (RCA1-1 and RCA1-2), 0.996 (RCA1-1 and RCA2), and 0.997 (RCA1-2 and RCA2), respectively.

To better identify low-frequency point mutations in mtDNA, we have developed a simple yet robust sequencing strategy, mitoRCA-seq (Fig. 1). Briefly, mtDNA is enriched from total genomic DNA by rolling circle amplification (RCA) using a set of custom mtDNA-specific primers in a single tube. The RELIP-g mitochondrial DNA kit can also be used as an alternative (See Methods for details) to perform the enrichment step for human and mouse samples. Since mtDNA is a circular molecule, it is preferentially amplified over the genomic DNA^21^. The resulting RCA products are then digested with a restriction enzyme, so that only full-length mtDNA can generate specific band(s). The mtDNA-specific fragments are gel purified (Supplementary Figure 1,2), and thus potential contamination of Numts is largely eliminated (Fig. 1A). The resulting mtDNAs are randomly sheared into ~300 bp fragments followed by ligation to Illumina adaptors (Fig. 1A, Supplementary Figures 1,2 and Methods). The final libraries were generated by either a PCR-free protocol or low-cycle PCR (Supplementary Figure 2, see Methods for details), and sequenced using Illumina platforms (HiSeq2000 and/or MiSeq). Compared to the traditional PCR-based mtDNA enrichment strategies^2^,^10^,^11^,^12^, the enrichment of mtDNA is achieved in a single reaction. In addition, fewer amplification errors are introduced during mitoRCA-seq library construction because RCA reaction is conducted with Phi29 DNA polymerase, a high-fidelity enzyme with an error rate of 1 in 10^6^–10^7^ bases^22^. Lastly, our proof-of-concept experiments showed that mitoRCA-seq can be successfully implemented with as little as 1 ng total genomic DNA (Supplementary Figure 1 and Supplementary Figure 9), thereby suitable for samples with limited quantity.

To assess the performance of mitoRCA-seq in detecting low-frequency mtDNA point mutations, we first estimated the baseline error rate resulting from mitoRCA-seq library construction and the sequencing process. Two independent PhiX (a 5,386 bp bacteriophage genome widely employed as a sequencing control) samples were sequenced with Illumina HiSeq2000 platform, acting as the control for evaluating sequencing errors. In addition, a 14,571 bp plasmid (pTEsindbisGFP, see Supplementary Table 1 for full sequence) of comparable length to that of mtDNA was subject to mitoRCA-seq except that plasmid-specific primers were used (Supplementary Table 1). Of the raw reads obtained, we first filtered out the sequence reads with an average sequencing quality score less than 30 (99.9% base-call accuracy). High-quality reads were then mapped to the reference plasmid sequence by BWA aligner^23^. To confidently distinguish the real mutation sites from the background noise, we compared the frequency distribution of single nucleotide variation (SNV) between controls (the plasmid and the PhiX) and real samples. The mutational spectrum of the plasmid and two PhiX controls were shown in Supplementary Figure 3. By applying our mutation-calling pipeline (see Methods for details), 14 mutations (out of 14,571 bases) were identified for the plasmid control with frequency greater than 0.3% (Supplementary Table 4). Under such criteria, the false discovery rate (FDR) is 0.00096 (14/14571 or <1 × 10^−3^). Notably, 13 out of the 14 mutations have an allele frequency close to 1, suggesting that they are not sequencing errors but true polymorphisms of the reference sequence. This would reduce the FDR to 7 × 10^−5^ (or 1/14571). Comparable results were obtained for PhiX controls (4 and 1 sites out of 5,368 bases, or FDR <1 × 10^−3^, see Supplementary Table 4). In addition, the mutation spectrums are considerably different among plasmid, mitochondria DNA from wild-type and mutant mice (Supplementary Figure 3). As expected, the mutation frequency is highest in *Polg* mutant mice and lower in plasmid control. Minor Allele Frequency (MAF) >0.3% was chosen as the cut-off since with such threshold, the error rate is relatively low (FDR <1 × 10^−3^ as shown both in the plasmid and the PhiX controls).

### High reproducibility and sensitivity of the mitoRCA-seq method

MitoRCA-seq was applied to analyze the *Polg* mutant mice, which were originally generated by Trifunovic *et al.* and purchased from Jackson laboratory^5^. The lack of error correction during mtDNA replication in the *Polg* mutant mice is expected to elevate the mutation load in mtDNA^1^,^5^. Overall, 16 mitoRCA-seq libraries were constructed for *Polg* mutant and corresponding wild-type mice. For each mouse strain, two tissues (brain and liver) were obtained from two littermates, and library constructions were carried out with both low-cycle PCR and PCR-free strategies starting with 100 ng total DNA (Supplementary Figure 2 and Supplementary Table 5 for detail).

**Figure 2:**
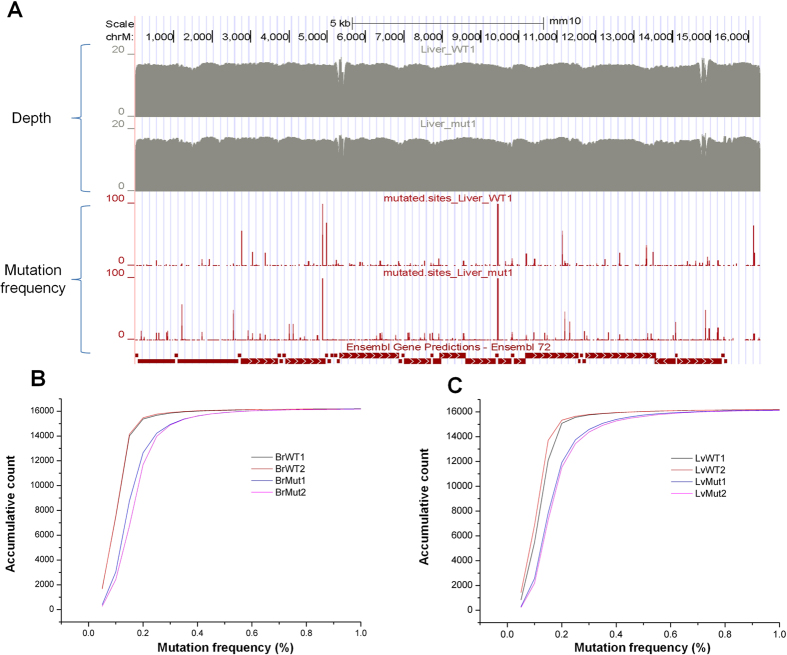
The mtDNA mutation profiles determined by mitoRCA-seq. (**A**) The evenness of mitoRCA-seq coverage are shown for two liver samples (upper panel). The coverage depth, or Log2 transformed uniquely mapped reads at a given nucleotide position (y axis), is plotted along the mouse mitochondrial genome (x axis). The frequencies of the mutations identified in the same samples are shown in two bottom panels. (**B**) and (**C**) are cumulative plots based on the frequency of mtDNA mutations identified in the brain (Br) and liver (Lv) samples. Four samples are shown for each tissue, consisting of biological replicates derived from two wild-type (WT1 and WT2) and two *Polg* mutant mice.

In order to reliably identify single nucleotide mutations, we devised a data analysis pipeline (Fig. 1B). We applied the same parameters (quality score ≥30; SNV frequency ≥0.3%; BUR ≥3; BUR frequency ≥0.2%; see Methods and Supplementary Figure 4 for details), which was determined based on the control experiment with plasmid DNA, for calling mitochondrial mutations. The use of BUR (best-unique reads) allows the incorporation of base quality scores into mutation identification. Overall, we obtained ~239 million high quality reads uniquely mapped to the reference mouse mtDNA (Supplementary Table 5), and >90% of the mouse mtDNA sequence was covered more than 1000 times for the PCR-based libraries (Supplementary Figure 5A). For PCR-free libraries, >50% of the bases were covered more than 1000 times (Supplementary Figure 5B). The relatively lower coverage in the latter libraries was due to lower sequencing depth. Our data showed relatively even coverage for all samples (Fig. 2A; Supplementary Figure 5,6). With an average depth of 52,496–55,780 fold for low-cycle PCR libraries and 1,220–7,651 fold for PCR-free libraries, it is sufficient to identify low-frequency point mutations as low as 0.3%. The corresponding read coverage and mutation frequencies across the whole mitochondrial genome are shown in Supplementary Figure 7. More importantly, the frequencies of point mutations identified by low-cycle PCR and PCR-free strategies are highly correlated (correlation coefficient R = 0.983~0.996, Supplementary Figure 8), indicating the low-cycle PCR step introduces little coverage bias and amplification errors.

In addition to biological replicates performed for each sample, we further evaluated the reproducibility of mitoRCA-seq by analyzing three technical replicates of a liver sample obtained from a *Polg* mutant mouse. Two of the technical replicates used the same RCA reaction for library construction, while the third library was constructed using an independent RCA reaction to determine whether RCA manipulation could affect mutation frequency. We observed a strong correlation among three technical replicates (R = 0.986~0.998, Fig. 1C). Taken together, these data demonstrated that mitoRCA-seq is a reliable method to detect low-frequency point mutations in mtDNA.

We next evaluated the quantity of total DNA required for mtDNA enrichment and library preparation. Total DNA from *Drosophila Melanogaster* was serial diluted from 100 ng to 0.1 pg. The results showed that RCA products derived from 1 ng of total DNA can generate a signature digestion pattern, an indicator of good signal to noise ratio for mtDNA enrichment (Supplementary Figure 1B). Based on this observation, three libraries, starting with 1 ng, 5 ng and 50 ng of total DNA isolated from mouse liver, were constructed and sequenced by Illumina MiSeq platform. While the percentage of raw reads mapped to mitochondrial genome gradually decrease as less DNA inputs were used, the on-target mapping rate of the 1 ng sample is still in the acceptable range (68%; Supplementary Table 6). In addition, the three samples show high concordance in frequency of SNV identified (R = 0.96~0.98, Supplementary Figure 9). Taken together, our data suggest that mitoRCA-seq can be adapted for as low as 1 ng of total DNA to detect low-frequency point mutation in mtDNA.

### MitoRCA-seq has less Numt contamination than PCR- and capture-based methods

It has been reported that Numts contamination presents a great challenge for reliable monitoring of mtDNA mutations, especially those occurring at low-frequency^11^. Since our RCA-based strategy specifically enriches full-length mtDNA fragments (Fig. 1A), it can largely minimize Numts contamination. To evaluate the performance of mitoRCA-seq compared to other strategies, we downloaded several publicly available datasets generated by long-range PCR or capture-based approach^11^,^24^. By applying the same stringent criteria to call Numts (see **Methods** for details), we found long-range PCR method has a contamination rate of 0.40–0.60% while the percentage of capture-based approach is 0.60–1.14%. The mitoRCA-seq has the lowest level of Numt contamination (0.19–0.33%, Supplementary Figure 10 and Supplementary Table 8). Thus, mitoRCA-seq serves as an improved method for identification of low-frequency mtDNA point mutation and is less influenced by Numt contamination.

### The pattern of heteroplasmic mtDNA mutations in *Polg* mice

**Figure 3:**
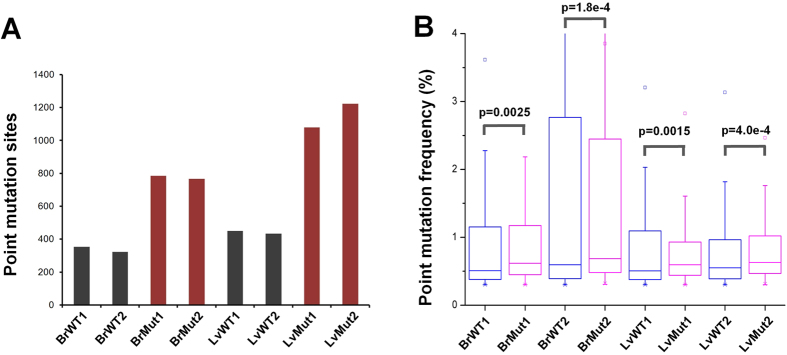
Comparison of the mutation load between wild-type and *Polg* mutant mice. (**A**) The number of mutation sites reliably detected in individual samples. (**B**) For each comparison, mtDNA mutations that are shared between a pair of wild-type and *Polg* mutant littermates in the given tissue were first identified. WT1 is the littermate control of Mut1, and both mice were generated from the same cross of heterozygous *Polg* mice. WT2 and Mut2 are littermates and served as biological replicates. The overall frequency of these shared mutations was then compared in the corresponding sample pairs (KS test).

Mutational load can be separated into the number of mutation sites and mutation frequency of individual sites. By applying a stringent strategy (see **Methods** for details) to discern low-frequency point mutations from sequencing background, we identified 208–460 putative mutation sites in wild-type mice and 516–1,381 sites in *Polg* mutant mice, respectively (see Supplementary Table 5 for details). Regardless of the library construction strategies between low-cycle PCR and PCR-free protocols, the mutator mice contain more mutation sites than wild-type mice (2.52–2.88 fold in brain and 3.27–3.40 fold in liver; Fig. 2 and 3A,B and Supplementary Table 5). In addition to newly acquired mutation sites, the mutant mice also show a slight increase in the frequency of existing mutation sites shared with wild-type mice (p value <0.003, KS-test, Fig. 3B and Supplementary Figure 11), thereby suggesting that the increase of mutation load in the *Polg* mutant mice is achieved through multiple fronts. Ameur *et al.* showed that homozygous *Polg* mutant mice have a higher number of mutation sites (~5.3–6.6 times) compared with wild-type mice at the age of 30–40 weeks^1^, while we found the number of mutational sites increase ~3 times in 6-week old *Polg* mutant mice compared to age matched controls. Consistent with our finding, Trifunovic *et al.* reported that younger *Polg* mutant mice has less mutations per 10 kilobase than older ones, although the study was based on Sanger sequencing of specific regions of mtDNA (the *Cytb* gene and control region)^5^. Taken together, it implies that mutations are gradually accumulated in *Polg* mutant mice long before the detectable phenotypes.

**Figure 4:**
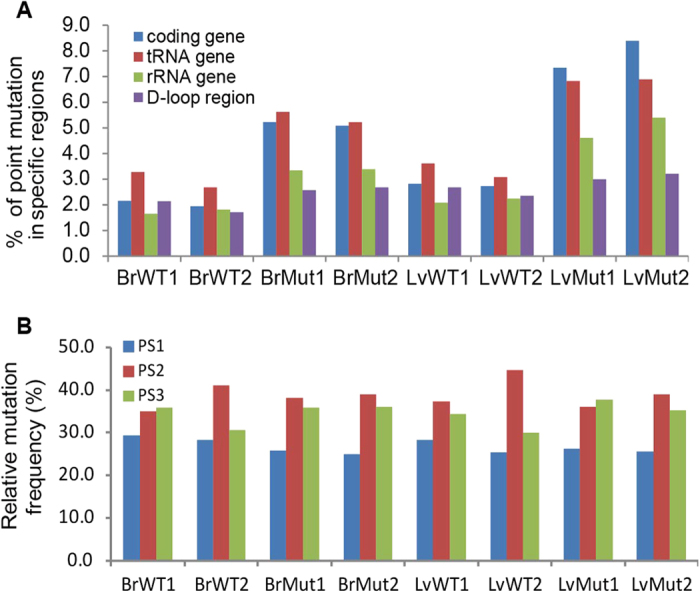
The location preference of heteroplasmic mtDNA mutations. (**A**) The proportion of point mutations identified in individual mitochondrial regions. The percentage was calculated by dividing the number of mutation sites with the total number nucleotides of each mtDNA region of interest. (**B**) The relative frequency of mitochondrial mutations at different codon positions for mutations located in the protein-coding genes. PS1, PS2 and PS3 refer to the relative positions of a triplet genetic codon.

The mitochondrial genome encodes a handful of protein-coding genes and functional RNAs (transfer and ribosomal RNAs). The remaining sequence is largely known as D-loop region. A comparison of wild-type and *Polg* mutant mice showed that more mutation sites are introduced to the protein-coding and tRNA genes, whereas the D-loop region is the least affected (Fig. 4A). Similar trend was also detected for mutation load, which is calculated by integrating mutation sites and their observed frequencies (Supplementary Table 7). Our result that D-loop region has less mutational load compared with other regions in *Polg* mutant mice is consistent with Ameur *et al.*’s study, which showed that the mutational load of D-loop region is 59–66% lower than protein coding region in 30–40 week old *Polg* mutant mice^1^. However, Ameur *et al.*’s study didn’t compare the mutation sites between D-loop region and other regions (e.g. protein coding and rRNA regions). In contrast, Kennedy *et al.* reported in human normal aging brains that D-loop region exhibits an elevated mutation load compared with protein coding regions^25^. Although we cannot rule out species-specific location preference in mtDNA mutations (human vs. mouse), these results imply that normal aging and *Polg*-dependent premature aging involve different mechanisms of mutation accumulation in D-loop region. Since protein-coding genes comprise a large proportion (70%) of the mtDNA genome, we next examined the location of mutant sites in regard to codon positions, and no mutation positional preference can be detected (Fig. 4B). This result is consistent with a previous study on *cytochrome b*^5^, suggesting that mtDNA mutations are not biased to any codon position in particular.

### Unbalanced cytocine (C) to thymine (T) transition in *Polg* mutant mice

**Figure 5:**
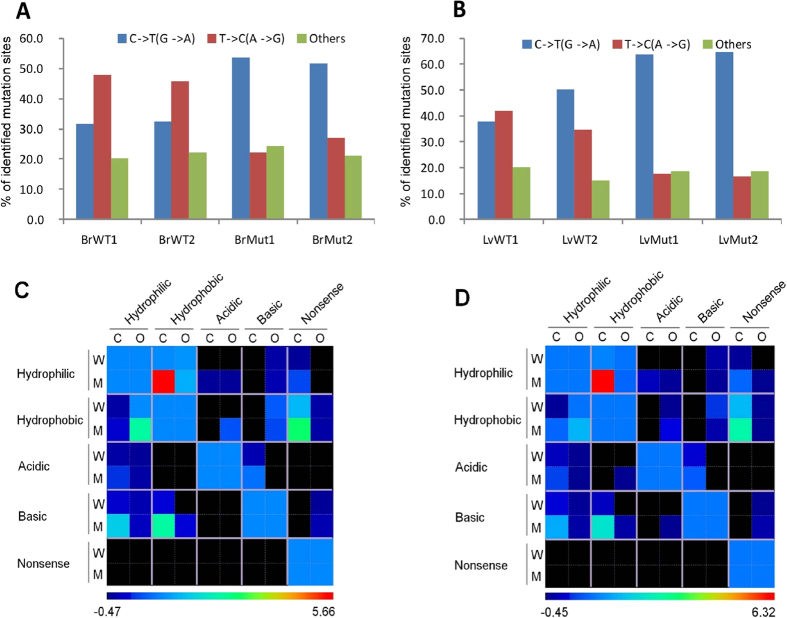
C → T transitions are overrepresented in the *Polg* mutant mice. (**A**) and (**B**) The percentage of transition (C → T and T → C) and transversion mutations detected in individual samples derived from mouse liver and brain respectively. (**C**) and (**D**) C → T transitions identified in *Polg* mutant mice tend to increase the hydrophobicity of corresponding amino acids. Briefly, C → T transitions were compared with other types of mutations (O) in their ability to alter amino acid property. For individual mutations identified by mitoRCA-seq, the properties of the corresponding amino acids in the reference genome are shown in rows, and the amino acid properties resulting from individual mutations are shown in column. Enrichment scores (Z-score) were then computed for all possible combinations. For each biological replicate of a given tissue, enrichment analyses were performed separately for wild-type (W) and mutant (M) mice, and the results are shown in a combined heat map. Two plots are shown based on the results derived from the brain (**C**) and liver (**D**) of the wild-type (WT1) and *Polg* mutant (Mut1) mice.

The pattern of base substitutions (transitions and transversions) was next examined for the mutations identified by mitoRCA-seq. We focused on the low-cycle PCR libraries because they have higher coverage than PCR-free libraries, and the results between the two approaches are highly correlated (R = 0.983~0.996, Supplementary Figure 8). Among all possible transitions and transversions, cytosine (C) to thymine (T) transition (or G to A on the complementary strand) is more prominent in the *Polg* mice compared to the control mice (Pearson’s Chi-squared test, p value <2.2 × 10^−16^ for both liver and brain), accounting for more than half of all base substitutions in *Polg* mutant mice (Fig. 5A,B). In addition, *Polg*-specific mutations consist of 42.4–50.9% C → T transitions, while the percentage for wild-type-specific mutations is 8.5–10.9% (Supplementary Table 9). Moreover, T → C mutations showed the opposite trend, 8.3–14.4% for *Polg*-specific mutations and 34.0–40.3% for wild-type-specific mutations, respectively (Supplementary Table 9). The unbalanced base substitution pattern agrees with a recent study based on an ultra-sensitive duplex sequencing technique^26^. Notably, C → T transition can lead to wobble base pairing (G·T) where the original cytosine (which is paired with G) is replaced by a thymine. Because G·T pairing has comparable thermodynamics to canonical base pairing^27^, it is conceivable that the prevalence of C → T transition in *Polg* mice may result from a defect in correcting G·T base pairing during mtDNA replication.

### C to T transition increases the hydrophobicity of amino acids encoded

Since most of the mtDNA mutations (70%) occur at protein-coding loci, we next examined how these mutations may affect the property of corresponding amino acids. In particular, we focused on C → T (G → A) transitions because they are the predominant base substitutions in *Polg* mutant mice (Fig. 5A,B). To this end, we first simulated all possible C → T (G → A) transitions in the coding regions. Of them, 44.7% (1903/4255) could lead to a change in amino acid property. In contrast, 55.1–74.4% of C → T (G → A) mutations identified by mitoRCA-seq alter the property of the respective amino acids. These results suggested that the low-frequency C → T (G → A) transitions accumulated in the mitochondrial genome might be more deleterious as more amino acids are affected than expected from genome-wide background. Comparison between C → T (G → A) transition and other types of base substitutions showed that changes from hydrophilic to hydrophobic amino acid are overrepresented in *Polg* mutant mice, while the trend is not significant in wild-type mice (Pearson’s Chi-squared test, p value = 0.0034 for liver and 0.0154 for brain, Fig. 5C,D, Supplementary Figure 12, Supplementary Table 10). It is possible that the prevalence of C → T transitions and the accumulation of peptides with altered function may ultimately contribute to the premature aging phenotype of *Polg* mutant mice.

### C to T transition has strand bias

**Figure 6:**
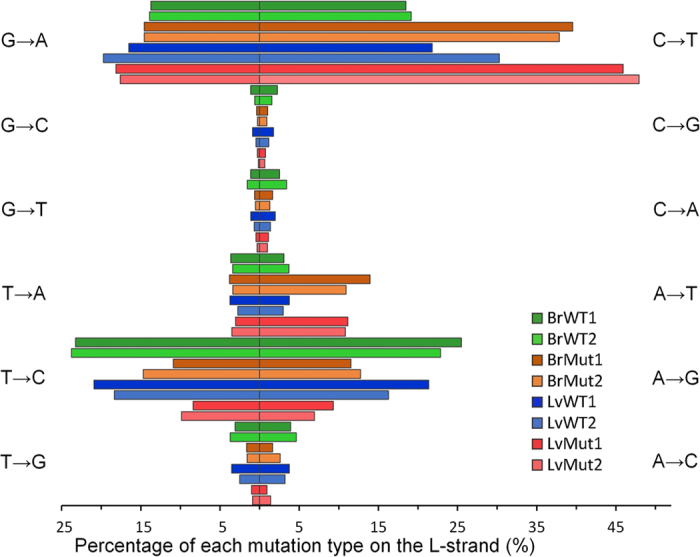
The strand asymmetry of C → T transitions in *Polg* mutant mice. For mutation identification mitoRCA-seq reads were mapped to the L-strand of the reference mouse mitochondrial genome. The resulting mutations were subdivided into all possible 12 categories, and the percentages of mutations detected in each reciprocal pair (e.g., C → T and G → A) are plotted side-by-side horizontally. In both *Polg* mutant mice C → T transitions are much higher than G → A transitions (or C → T transitions on the H strand), indicating stand asymmetry of C → T transitions in the mutator mice.

The mitochondrial genome consists of two strands, heavy strand (H strand) and light strand (L-strand or the reference sequences in public databases). Interestingly, C → T transitions are more frequent than G → A transitions in wild-type mice on the L-strand (on average 1.36-fold in brain and 1.43-fold in liver, Fig. 6). The mutation asymmetry or strand bias is more pronounced in *Polg* mutant mice than wild-type mice (on average 2.66-fold in brain and 2.63-fold in liver, Pearson’s Chi-squared test, p value <0.004, Fig. 6). Our result is different from Kennedy *et al.*’s finding that C → T transitions accumulate on the H strand in aged human brain^25^. Such inconsistency might reflect the underlying difference of mtDNA mutation between human normal brain aging and mouse premature aging. We speculate that C → T transition during normal aging may be partially resulting from spontaneous base hydrolysis, for which the H-strand might be more susceptible^28^, possibly due to the asynchronous replication of mitochondrial genome^29^. C → T transitions accumulated in *Polg* mutant mice are likely resulting from G.T wobble base-pairing. It is expected to favor the L-strand because the H-strand contains more guanines than the L-strand.

### C to T transition has a trinucleotide context preference

**Figure 7:**
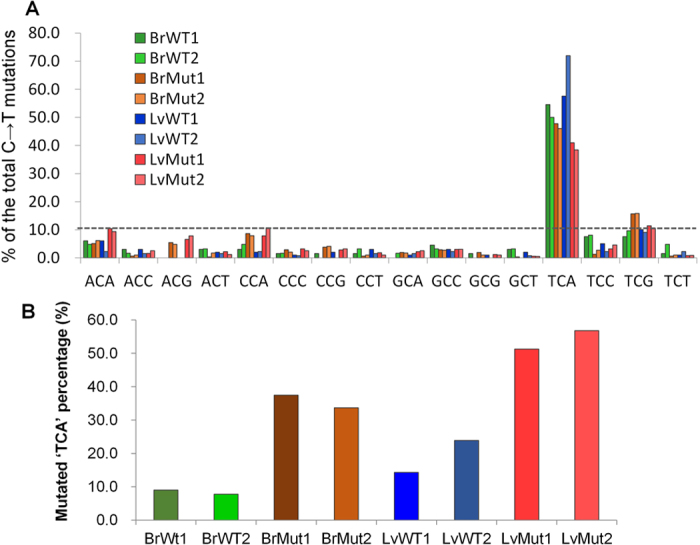
The context dependency of C → T transitions identified in mouse mtDNAs (**A**) The frequency of 16 all possible trinucleotides, in which the middle nucleotide is subject to C to T transition. The ratio of ‘TCA’ and ‘NCN’ trinucleotides found in the mouse mitochondrial genome is shown as a dashed line. ‘N’ denotes any nucleotide. (**B**) The percentage of ‘TCA’ trinucleotides in the mouse mitochondrial genome that contain a C → T transition in different samples.

Next we asked whether the local sequence context plays a role in defining the location of C → T transitions. To this end, we computed the frequency of all 16 possible ‘NCN’ trinucleotide combinations surrounding the C → T transitions identified. The results showed that the first nucleotide tends to be ‘T’ and the third nucleotide tends to be ‘A’ (Supplementary Figure 13). Notably, ‘TCA’ accounts for 38.4–72.0% of all mutated ‘NCN’ trinucleotides in both wild-type and mutant mice (Fig. 7A). In contrast, only 10.3% of the ‘NCN’ trinucleotides across the mitochondrial genome are ‘TCA’. These results strongly suggested that C → T transition is context dependent. The prevalence of C → T transition among all ‘TCA’ trinucleotides in the mitochondrial genome was next determined. Strikingly, C → T transition was observed in a large proportion (33.7–37.4% in brain and 51.3–56.8% in liver) of all TCA trinucleotides in the case of mutator mice (Fig. 7B). This observation is unlikely an experiment artifact because the fraction is considerably lower for wild-type mice (7.8–9.0% and 14–24% in brain and liver, respectively) (Fig. 7B). Although the molecular mechanism underlying the context dependency is largely unknown, our results provided a foundation for further investigation of the fidelity of mtDNA replication under physiological and pathological conditions.

## Discussion

Here we present a mitoRCA-seq method for detecting low-frequency mitochondrial mutations. The enrichment of mtDNA by RCA is carried out in a single tube with a set of mtDNA-specific primers (Fig. 1A), and thus does not require multiple PCR reactions or sophisticated instrumentations. As the result, full-length mtDNA fragments can be size selected and purified, and contamination of Numts is largely minimized (Supplementary Figure 10 and Supplementary Table 8). In addition, mitoRCA-seq results are highly reproducible between technical replicates (R = 0.986~0.998, Fig. 1C) and replicates between two protocols (PCR-free and low-cycle PCR, R = 0.983~0.996, Supplementary Figure 8). MitoRCA-seq was successfully tested for mouse and human (Supplementary Figure 1), and has the potential to be broadly applicable for diverse organisms by using species-specific mtDNA primers. Notably, our proof-of-concept experiments showed that as little as 1 ng of total DNA (equal to 100–200 cells) is sufficient for mtDNA enrichment and downstream library construction (Supplementary Figure 1, 9, Supplementary Table 6), therefore making mitoRCA-seq a possible approach analyzing samples with limited DNA quantity. Further experiments are warranted to determine the feasibility of mitoRCA-seq for patient samples in the clinical settings.

Detecting low-frequency mutations (e.g., heteroplasmic mtDNA mutations and somatic mutations in tumor samples) is one of the major challenges in Next Generation Sequencing analysis^1^,^2^,^11^,^14^,^17^. Erroneous bases can be introduced at multiple steps during library construction and sequencing^11^,^14^,^17^. Library preparation, especially PCR amplification, is likely to be the major source of false positives in mutation callings for both long-range PCR and capture-based approaches^14^,^30^. MitoRCA-seq only requires a low-cycle PCR amplification and even entirely eliminates the PCR step (PCR-free procedure). Therefore, it is expected to improve the sensitivity in mutation detection by reducing potential false positives. In addition, mitoRCA-seq can be combined with bidirectional duplex sequencing strategy to further reduce sequencing error in mutation identification^26^. Duplex sequencing employs randomized duplex tag in the adaptors, thereby allowing for the removal of sequencing errors by computational filtering. However, this strategy depends on isolation of mitochondria and requires greater than 100 ng of input materials^25^. MitoRCA-seq can start with as little as 1 ng of total DNA (Supplementary Figure 1,9, Supplementary Table 6), and the RCA reaction could tolerate total DNA isolated from paraffin embedded tissues^31^,^32^. Therefore, it makes mitoRCA-seq a potentially powerful approach to profile low-frequency mitochondrial point mutations in a variety of applications. Additional improvements can also be made for mitoRCA-seq, such as enhancing the mtDNA enrichment efficiency by eliminating linear DNA with exonuclease V digestion^33^ before RCA step.

By comparing mtDNA mutation profiles of wild-type and *Polg* mutant mice, we found that the overall mutation load of the mutator mice is increased by expanding the number of mutation sites (Fig. 2; Figs 3A,B; Supplementary Table 5), and to a lesser extent by elevating the mutation frequency at the existing sites (Fig. 3B and Supplementary Figure 11). Notably, C → T transition is overrepresented in the mutator mice compared to the normal controls (Fig. 5). Further analyses revealed that C → T transitions, if located in protein-coding regions, tend to alter the hydrophobicity of the amino acids encoded. As these data were obtained from 6-week mice, much earlier than ~25-week when the premature aging phenotypes become detectable^5^, our study thus indicates that molecular events related to possible protein and cellular functions have already changed in the *Polg* mutant mice before visible phenotypes appear. We found D-loop region has less mutation load compared with other regions in *Polg* mutant mice. Our finding is consistent with the study by Ameur *et al.*, who also employed *Polg* mutant mice as a model system^1^. In contrast, Kennedy *et al.* showed that D-loop region exhibits an increased mutation load compared with protein coding regions in human aging brains^25^. Williams *et al.* used Mito-Seq methodology to investigate mtDNA mutation spectra of putamen from young and aged donors^19^. They showed that point mutations are elevated with age with the highest rate of accumulation in the D-loop region. The discrepancies among these studies imply that normal aging in human and *Polg*-dependent premature aging in mice may involve different mutational mechanisms in the D-loop region.

MitoRCA-seq can also be used for detecting insertion/deletions (InDels). We focused on small InDels because the restriction digestion and size selection procedure is expected to bias against large InDels. The results showed that small InDels occur in a much lower frequency than single nucleotide point mutations (Supplementary Figure 14). In addition, the majority of the small InDels are less than 4 nucleotides, with one nucleotide as the dominant group (Supplementary Table 11). Furthermore, there were increased numbers of small InDels in *Polg* mutant mice than wild-type controls (Supplementary Table 11), consistent with the previous finding that mutator mice have higher level of small InDels^1^.

We speculate that wobble base pairing (G·T), which has comparable thermodynamics to canonical base pairing^27^, contributes to the prevalence of C → T transition in *Polg* mice due to the defect in correcting G·T base pairings during mtDNA replication. Kennedy *et al.* provided evidence that replication errors by DNA polymerase γ, or spontaneous base hydrolysis, but not oxidative damage, are the major driver(s) for the elevated point mutations detected in mitochondrial DNA during human normal aging^25^. These studies highlight the importance of replication error in generating low frequency point mutations in mtDNA. In addition to replication error, other mechanisms, e.g., cytosine deamination, may also contribute to the C → T transitions^34^. Notably, existing mutations in mtDNA can be eliminated as well, possibly through purifying selection during germ line transmission and/or selective turnover of dysfunctional mitochondria by autophagy^35^. It is conceivable that mtDNA mutations are highly dynamic depending on the physiological and/or pathological states of the cell. Therefore, a reliable strategy for profiling mtDNA mutations, such as mitoRCA-seq, is expected to promote a better understanding of mtDNA mutation in human diseases.

## Methods

### DNA extraction from mouse tissues

All experiments were performed in accordance with relevant guidelines and regulations. All animal procedures followed the US National Institutes of Health guidelines Using Animals in Intramural Research and were approved by the National Institutes of Mental Health Animal Care and Use Committee. Brain and liver tissues were taken from 6-week old wild-type and *Polg* mutant mice (C57BL/6). Each mutant mouse has a wild-type littermate control, both of which were generated from the same cross of heterozygous *Polg* mice. Two pairs of wild-type and mutant mice were used serving as biological replicates. QIAamp DNA Blood Mini kit (Qiagen) was used to extract total DNA from mouse tissues according to the manufacturer’s protocol. Briefly, 200 μl AL buffer was added to liver or brain sample (~20 mg) and the tissues were homogenized with one steel bead in a 2 ml tube with a Qiagen TissueLyser for 2 min at 50 oscillations per second. After column purification, total genomic DNA was eluted with 200 μl nuclease-free water and the DNA concentration was quantified with a Nanodrop spectrophotometer (Thermo Fisher).

### RCA with mitochondrial-specific oligonucleotides

For constructing mitoRCA-seq library, 100 ng of total DNA was used as template for RCA amplification. Briefly, a 50 μl reaction mix containing 100 ng total DNA, 1× Phi29 buffer (NEB), 0.2 μg/ml BSA, 1 mM dNTP (NEB) and 25 μM specific oligos (20 primer pairs for the pTEsindbisGFP plasmid, 19 primer pairs for mouse mtDNA and 13 primer pairs for *Drosophila* mtDNA) was denatured for 3 min at 95 °C. After cooling down at room temperature for 10 minutes, 1 μl of Phi29 DNA polymerase (10 unit/μl, NEB) was added to the reaction mix, which was then incubated at 37 °C for 16 hours followed by 65 °C for 10 minutes to heat inactivate the enzyme. For the titration test in Supplementary Figure 1, the input DNA amounts were titrated from 100 ng to 0.1 pg. The REPLI-g Mitochondrial DNA kit (Qiagen), which is based on RCA amplification, can also be used to selectively enrich human and mouse mtDNAs from total genomic DNA.

### Restriction enzyme digestions

Different restriction enzymes were selected to digest the RCA products according to the species’ mtDNA reference sequence to generate two distinguishable bands in 0.5% agarose gel. For mouse, EcoRV (NEB) was used to digest RCA products into two long fragments (9.5 kb and 6.8 kb). For human, SacI (NEB) was used to digest RCA products into two long fragments (9.6 kb and 6.9 kb). For fruit fly, NdeI (NEB) and EcoRV (NEB) were used to digest RCA products into two long fragments (10 kb and 9.8 kb). For plasmid pTEsindbisGFP, EcoRV (NEB) was used to digest RCA products into two long fragments (10.4 kb and 4.1 kb). All digestion reactions were carried out according to the manufacturer’s protocol.

### MitoRCA-seq library construction

Size selection of mitochondrial specific DNA fragments was carried out with 0.5% agarose gel. Zymoclean large fragment gel purification kit (ZYMO research) was used to purify the excised bands. The resulting DNA fragments were then sheared by Covaris S2 instrument into small fragments with a size peaked at 300 bp. End-IT kit from Epicentre was applied to perform end repairing, followed by size selection for 200–400 bp in a 2% agarose gel. A-tailing of the blunt-ended DNA was accomplished with Klenow exo- DNA polymerase (Epicentre). Illumina index adaptors were then ligated, followed by 8–12 cycle PCR with Phusion High Fidelity DNA polymerase (NEB). Size selection (300–500 bp) of the final PCR products was performed in a 2% agarose gel to prepare the mitoRCA-seq libraries, which were subjected to deep sequencing by Illumina HiSeq2000 or MiSeq instrument at DNA Sequencing and Genomics Core of NHLBI or Genergy Biotech (Shanghai) Co., Ltd. For PCR-free libraries, we followed the library construction protocol of NuGEN Encore Rapid Library Systems after the fragmentation step.

### MitoRCA-seq starting with limited DNA

To determine the minimum amount of total DNA required for mitoRCA-seq library construction, RCA reactions were set up with 100 ng, 1 ng, 10 pg or 0.1 pg genomic DNA. *Drosophila* genomic DNA and EcoRI (NEB) were used, which yields four distinct fragments to evaluate the enrichment of mtDNA fragments by RCA.

### Sequencing data analysis and mutation detection

Raw sequencing reads were first filtered by their average quality score. The sequence reads with an average quality score greater than 30 (99.9% base call accuracy) were kept for further analysis. The quality-filtered reads were aligned to the reference mitochondrial genome (UCSC, mm10) by BWA using default parameters and the uniquely mapped reads were used for further analysis. The mpileup package of SAMtools^18^,^36^ was used for the sequence variation discovery with the default parameters except with the *–d 200,000* to accommodate the actual sequencing depth. To remove the artificial variants caused by the sequencing errors, the mapping quality score at a point mutation site was set to greater than 30. To remove PCR artifacts, which tend to generate the same fragments and lead to duplicate reads in the final library, only one read of the best average quality (best-unique reads, BUR) was kept among all duplicated reads. The best-unique reads also pass through the same analysis pipeline as the quality-filtered reads. Briefly, any putative point mutation site must meet the following criteria: i) the nucleotide supporting the mutation should have a sequencing and mapping score greater than 30; ii) has a mutation frequency greater than 0.3%; and iii) the mutation site should also be supported by three or more BURs and mutation frequency supported by best-unique reads should be >0.2% to avoid sporadic sequencing errors due to higher coverage. The cutoff stringency of ≥3 BURs is adjusted based on average BUR coverage depth (Supplementary Figure 4).

### Calculate overlapping between Numts and mtDNA

To determine the potential nuclear mitochondrial like fragments (Numts) in the genome, the mouse mitochondrial genome (chrM, mm10) was aligned to the mouse nuclear genome (mm10) by BLASTn (NCBI Blast-2.2.26) program using the default parameters. All hits with length over 50 bp were kept as Numts fragments. A total of 143 Numts were defined in this way, with about 2.5× of the mitochondrial genome in size. *Diff-seq* package of *EMBOSS* suite of 6.5.7 version was used to identify single nucleotide variations between mitochondrial genome and the candidate Numts. Since the datasets from Li *et al.*^11^ are from human samples, we used human mtDNA (NC_012920.1) and human genome (hg19) for the alignment. To evaluate the possible contamination of reads from Numts, we aligned the Q20-filtered reads to Numts with BWA 0.6.1^19^,^23^. Reads with perfect match to Numts were extracted out and aligned to the mitochondrial genome. After filtering out reads with a perfect match to the mitochondrial genome, the remaining reads were considered as contaminations from Numts.

### Functional analysis of point mutations in mtDNA

To evaluate the functional consequence of mitochondrial point mutations, each mutation was assigned to the corresponding mitochondrial gene annotated in Ensemble database (GRCm38). The number of mutations in different regions, such as protein-coding genes, tRNA/rRNA genes and the regulatory region (D-loop), were calculated accordingly. For each mutation in the protein-coding gene the potential codon change was determined according to the vertebrate mitochondrial code from NCBI (https://www.ncbi.nlm.nih.gov/Taxonomy/taxonomyhome.html/ index.cgi? chapter=tgencodes#SG2). The amino acid property table (see Supplementary Table 8 for details) was used to assess the effect of individual mutations on amino acid properties.

## Additional Information

Accession codes: MitoRCA-seq raw data can be accessed at the NCBI Sequence Read Archive (SRP056905). 

**How to cite this article**: Ni, T. *et al.* MitoRCA-seq reveals unbalanced cytocine to thymine transition in *Polg* mutant mice. *Sci. Rep.* **5**, 12049; doi: 10.1038/srep12049 (2015).

## Supplementary Material

Supplementary Information

Supplementary Data
