# Supplementary material for: MitoRCA-seq reveals unbalanced cytocine to thymine transition in Polg mutant mice
Source: Sci Rep. 2015 Jul 27;5:12049. doi: 10.1038/srep12049 (PMC4648470; doi:10.1038/srep12049)
Supplement: Supplementary Information [file srep12049-s1.doc]

**MitoRCA-seq reveals unbalanced cytocine to thymine transition in *Polg* mutant mice**

Ting Ni, Gang Wei, Ting Shen, Miao Han, Yaru Lian, Haihui Fu, Kang Tu, Yanqin Yang, Jie Liu, Yoshi Wakabayashi, Zheng Li, Toren Finkel, Hong Xu, Jun Zhu

**Supplementary figures, tables and methods:**

| Supplementary Figure 1 | Estimation of the minimum DNA quantity required for constructing mitoRCA-seq library for mouse (A), fruit fly (B) and human (C,D). |
| --- | --- |
| Supplementary Figure 2 | Agarose gel images for key steps of mitoRCA-seq library construction for 8 mouse samples. |
| Supplementary Figure 3 | Estimation of background errors resulting from library construction and sequencing. |
| Supplementary Figure 4 | The coverage distribution of best-unique reads (BURs) among individual mitoRCA-seq libraries. |
| Supplementary Figure 5 | A summary of base coverage among individual mitoRCA-seq libraries. |
| Supplementary Figure 6 | A graphic view of coverage depth of individual libraries along the entire mitochondrial genome. |
| Supplementary Figure 7 | A graphic view of the mutation frequencies determined by individual mitoRCA-seq libraries. |
| Supplementary Figure 8 | The SNV frequencies determined by PCR-free and low-cycle PCR procedures are highly correlated. |
| Supplementary Figure 9 | The SNV frequencies are highly correlated between low and high input materials. |
| Supplementary Figure 10 | Comparison of Numt contaminations among different mitochondrial sequencing methods. |
| Supplementary Figure 11 | Comparison of the mutation frequency between wild-type and Polg mutant mice. |
| Supplementary Figure 12 | The effect of mitochondrial mutations on altering amino acid property. |
| Supplementary Figure 13 | The context dependence of the C→T transitions identified in the mouse mitochondrial genome. |
| Supplementary Figure 14 | Cumulative plots of SNP and small InDel mutations in brain (A) and liver (B). |
| Supplementary Table 1 | Primers used for constructing plasmid RCA-seq library. |
| Supplementary Table 2 | Primers used for constructing mouse mitoRCA-seq library. |
| Supplementary Table 3 | Primers used for constructing fruit fly mitoRCA-seq library. |
| Supplementary Table 4 | Counts of sites with background error frequency in the control samples. |
| Supplementary Table 5 | Summary of data analysis results based on Figure 1b. |
| Supplementary Table 6 | Mapping summary of data from 1 ng, 5 ng and 50 ng of mouse liver total DNA. |
| Supplementary Table 7 | Mutational load in D-loop region compared with other regions. |
| Supplementary Table 8 | Comparison of reads contamination from Numts. |
| Supplementary Table 9 | The relative abundance of C→T transitions in individual samples. |
| Supplementary Table 10 | Definition of amino acid’s property. |
| Supplementary Table 11 | *Polg* mutant mice have higher level of small InDels than wild-type mice. |
| Supplementary Methods | Detailed library construction methods and analyses methods |

**Supplementary Figures**

**A**


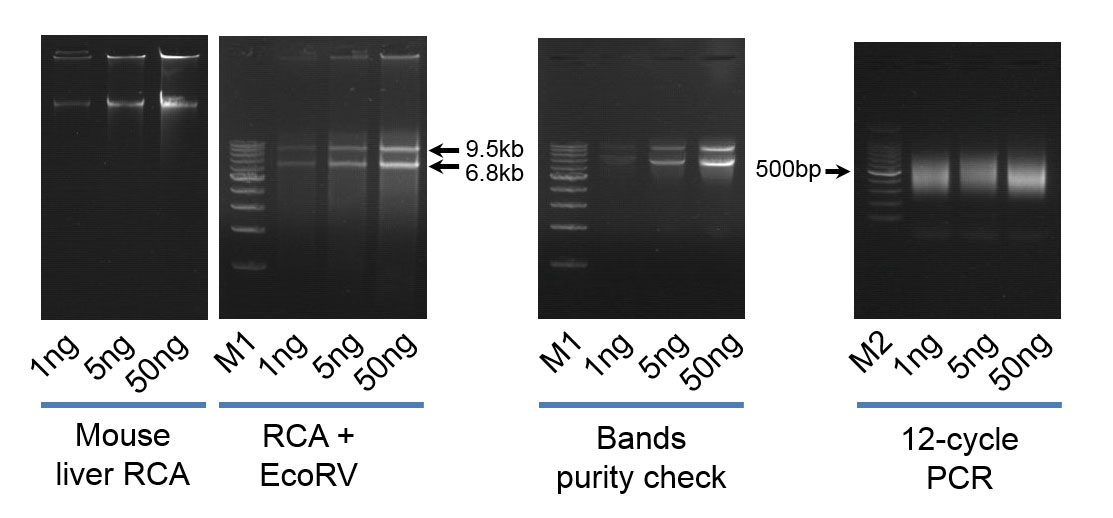


**B**


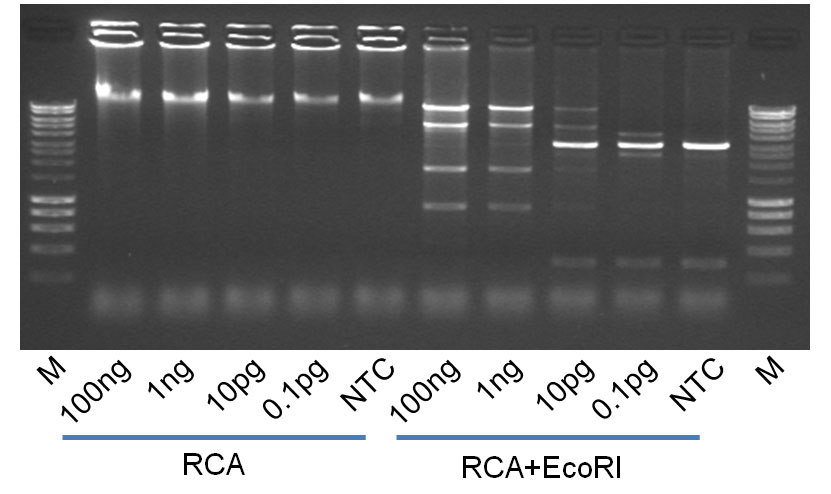


*****

*****

*****

**C**


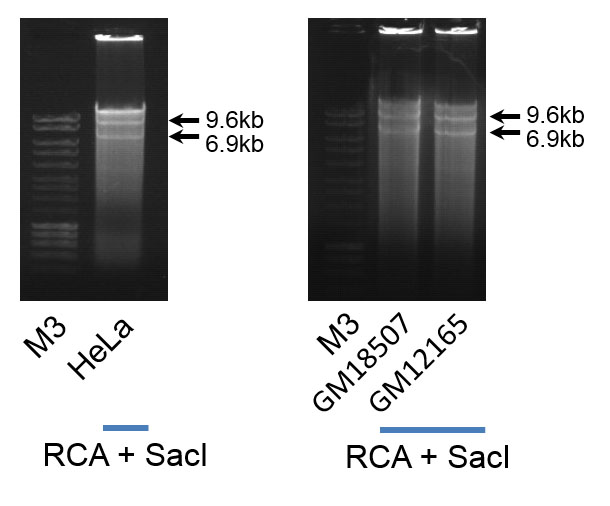


**D**


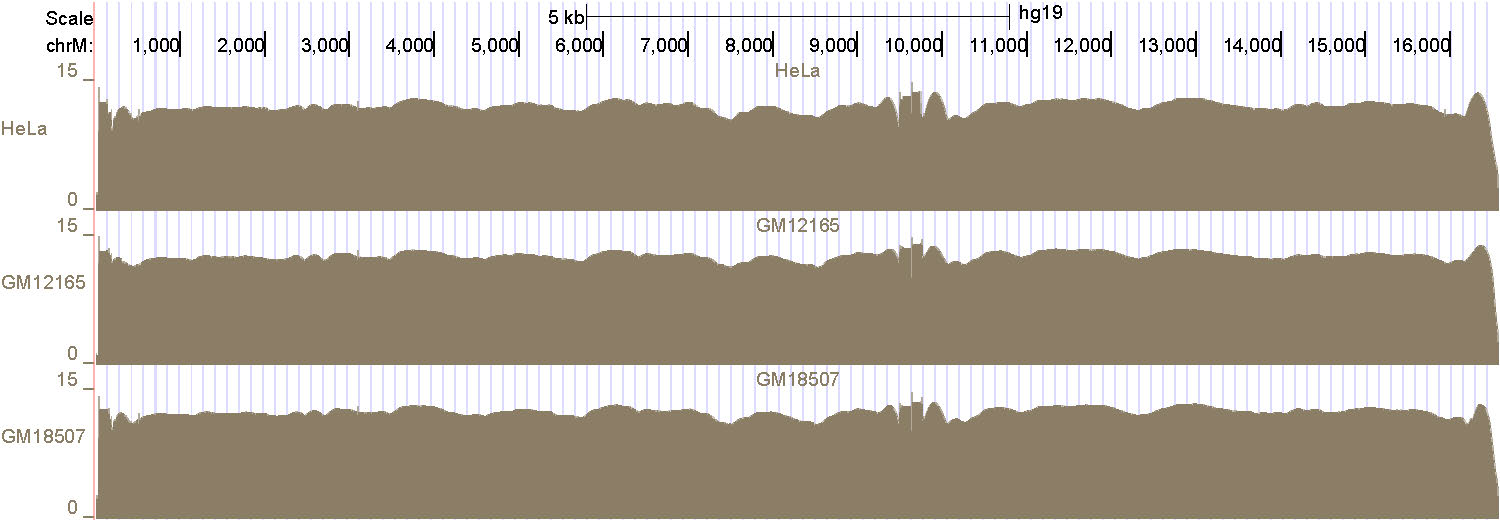


Supplementary Figure 1. Estimation of the minimum DNA quantity required for constructing mitoRCA-seq library for mouse (A), fruit fly (B) and human (C,D). (A) Different amounts of total DNA (50 ng, 5 ng and 1 ng) isolated from wild-type mouse liver were used as template for RCA using REPLI-g Mitochondrial DNA kit (Qiagen). The resulting RCA products were digested with EcoRV, which give rise to two discrete bands (9.5kb and 6.8kb) for full-length mtDNA substrates. After cutting and purifying the two bands from the agarose gel, the purity of the bands were further checked. We constructed the mitoRCA-seq libraries with 12-cycle PCR and sequenced them in MiSeq instrument for further evaluation. M1 and M2 denote two size markers suitable for different range from NEB. (B) Different amounts of total DNA (100 ng, 1 ng, 10pg and 0.1 pg) isolated from whole fruit fly were also used for RCA with Drosophila specific primers (see Supplementary Table for sequences). The resulting RCA products were digested with EcoRI, which give rise to four discrete bands for full-length mtDNA substrates. The signature digestion pattern can be detected for the RCA products derived from as little as 1ng of total genomic DNA, indicating 1 ng of total DNA from fruit fly is sufficient for the specific enrichment of mtDNA out of total DNA. NdeI and EcoRV double digestion, which generates two specific bands, was recommend for library construction of fruit fly ‘M’: Size marker (HyperLadder I, Bioline). ‘NTC’: No Template Control, in which nuclease-free water was used as template. * indicates digestion product of non-specific amplification that might derived from environmental contamination. (C) 100 ng of total DNA from three human cells were RCA amplified and digested by SacI to generate two distinct bands (9.6kb and 6.9kb). HeLa means total DNA derived from HeLa cell line, GM18507 and GM12165 denote total DNA of lymphoblastoid cells from two donors of the International HapMap Project. (D) A graphic view of coverage depth of individual libraries along the entire mitochondrial genome for the human samples. The coverage depths of individual mitochondrial bases (x-axis) are log2 transformed and shown on the y-axis.

**
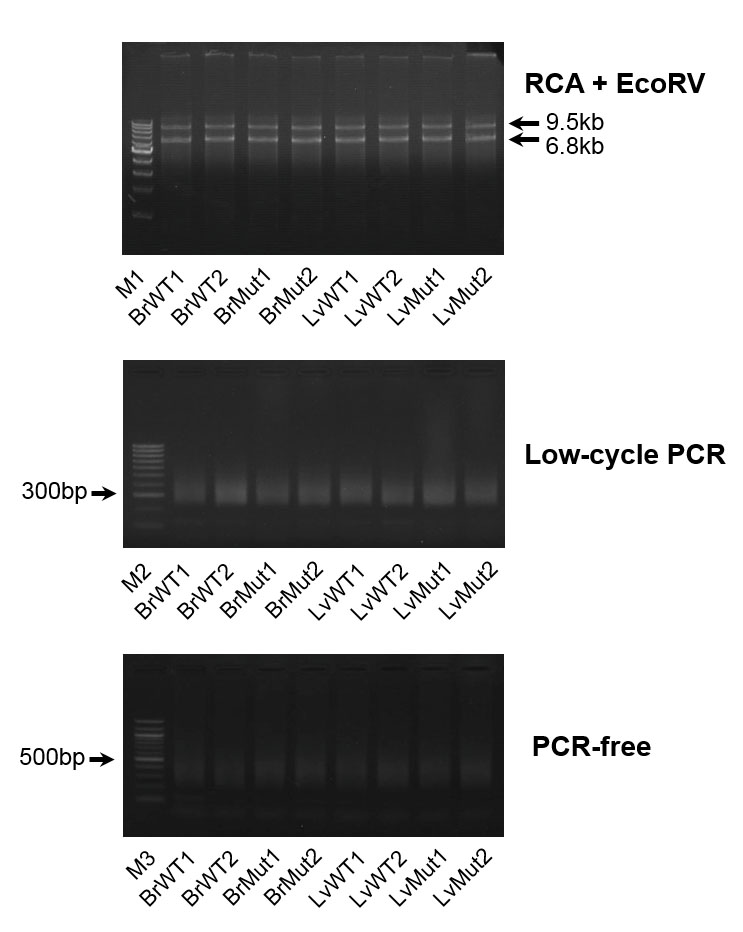
**

Supplementary Figure 2. Agarose gel images for key steps of mitoRCA-seq library construction for 8 mouse samples. The resulting RCA products were digested with EcoRV, which give rise to two discrete bands (9.5kb and 6.8kb) for full-length mtDNA substrates (labeled RCA + EcoRV). Two wild-type mice (WT1 and WT2) and two *Polg* mutant mice (Mut1 and Mut2) were investigated. Br and Lv represent Brain and Liver, respectively. After cutting and purifying the two bands from the agarose gel, two types of libraries (labeled Low-cycle PCR and PCR-free) were constructed and sequenced. M1, M2 and M3 represent DNA size markers suitable for different range from NEB.


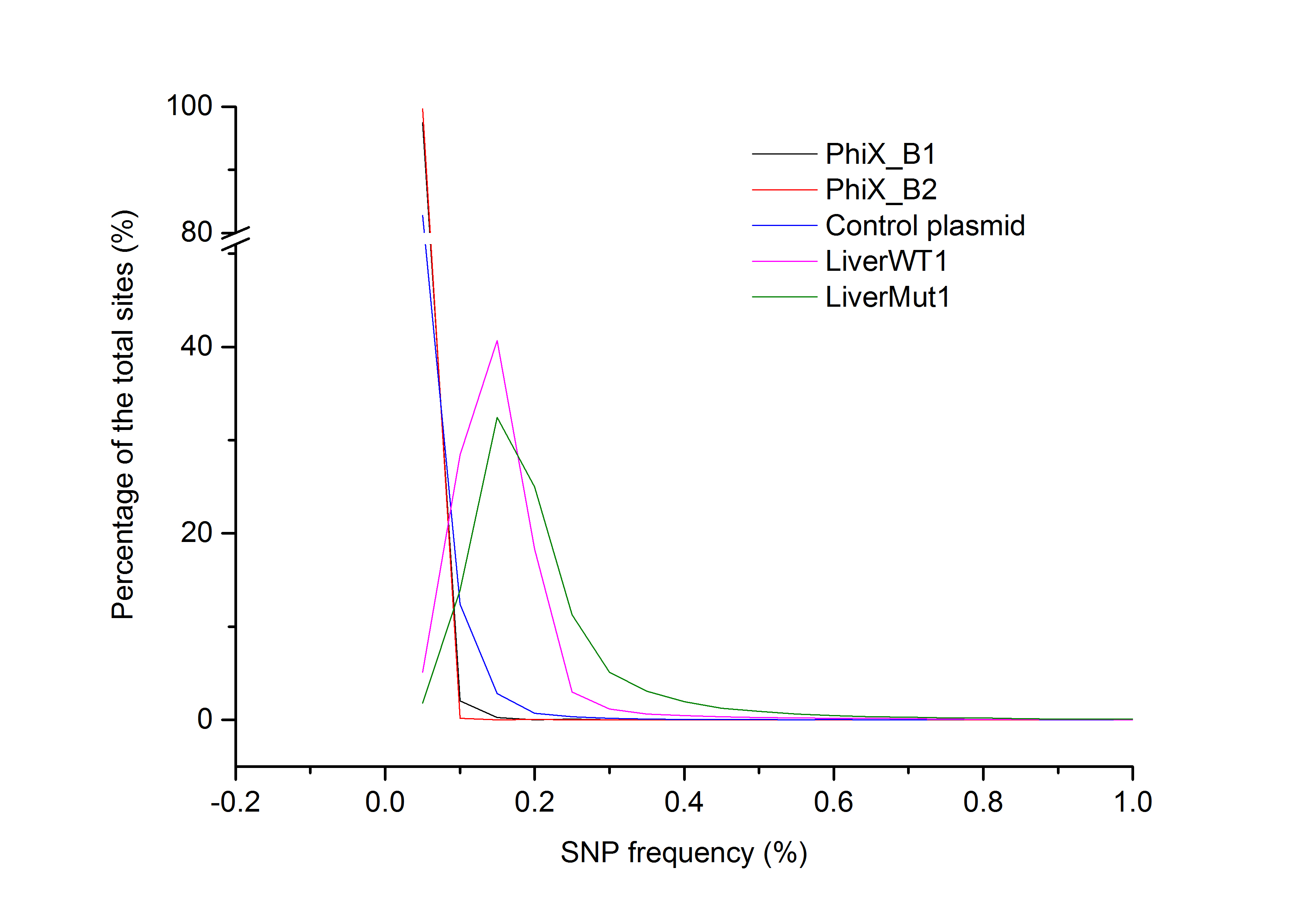


Supplementary Figure 3. Estimation of background errors resulting from library construction and sequencing. The X-axis represents the frequency of low abundant single nucleotide variation (SNV, 0.0%~1.0%), and the Y-axis shows the percentage of the mutated sites of a given frequency. More mutations were found in the liver of wild-type and *Polg* mutant mice compared to the controls, the pTEsindbisGFP plasmid and PhiX (two independent replicas: B1= Batch1 and B2 = Batch2) samples. In addition, the mutation distribution of mutant mice showed a right shift compared with that of wild-type mice, suggesting that more mutation sites at a higher frequency can be found in the *Polg* defective animal. MAF of 0.2% and 0.3% was shown in dashed lines.


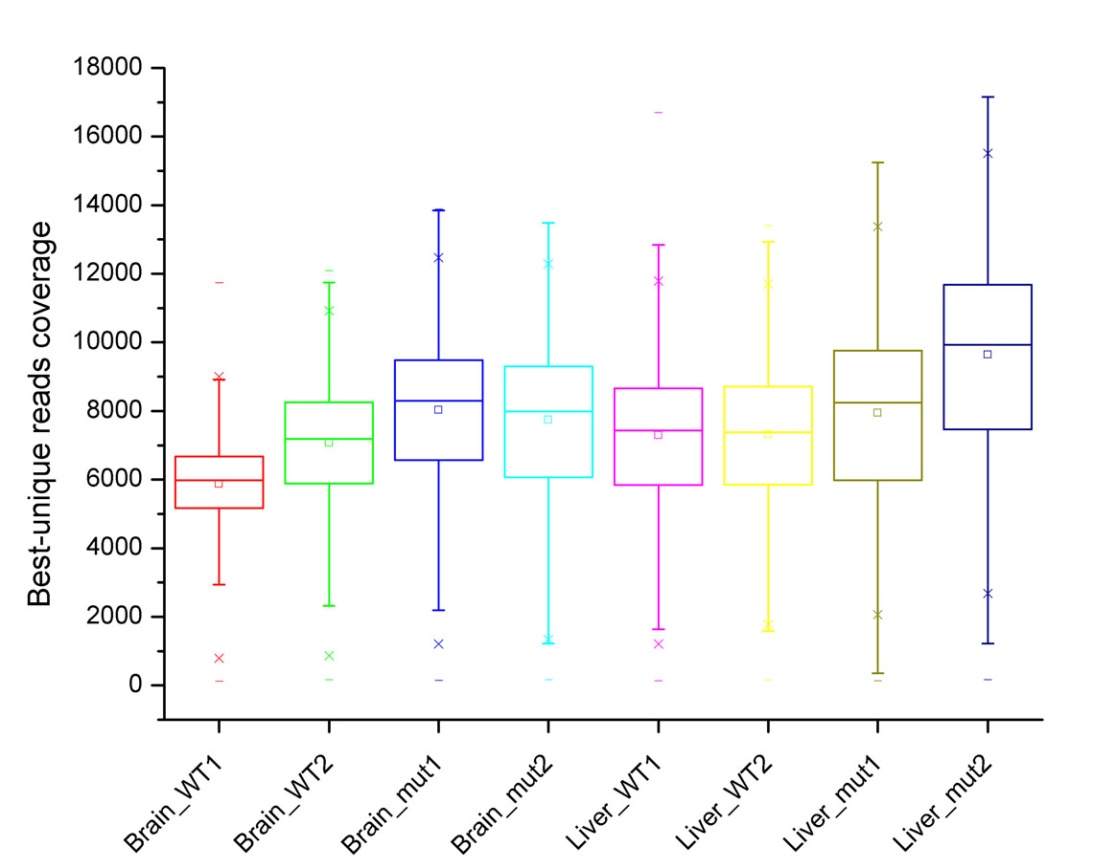


Supplementary Figure 4. The coverage distribution of best-unique reads (BURs) among individual mitoRCA-seq libraries. For duplicated reads that pass quality filter (average quality score >= 30), only one read with the best average quality (best-unique read) was kept. The coverage distribution based on the best-unique reads of each library is presented as a box plot. MitoRCA-seq was performed for two wild-type mice (WT1 and WT2) and two *Polg* mutant mice (mut1 and mut2). In each mouse, two tissues (brain and liver) were used for library preparation.


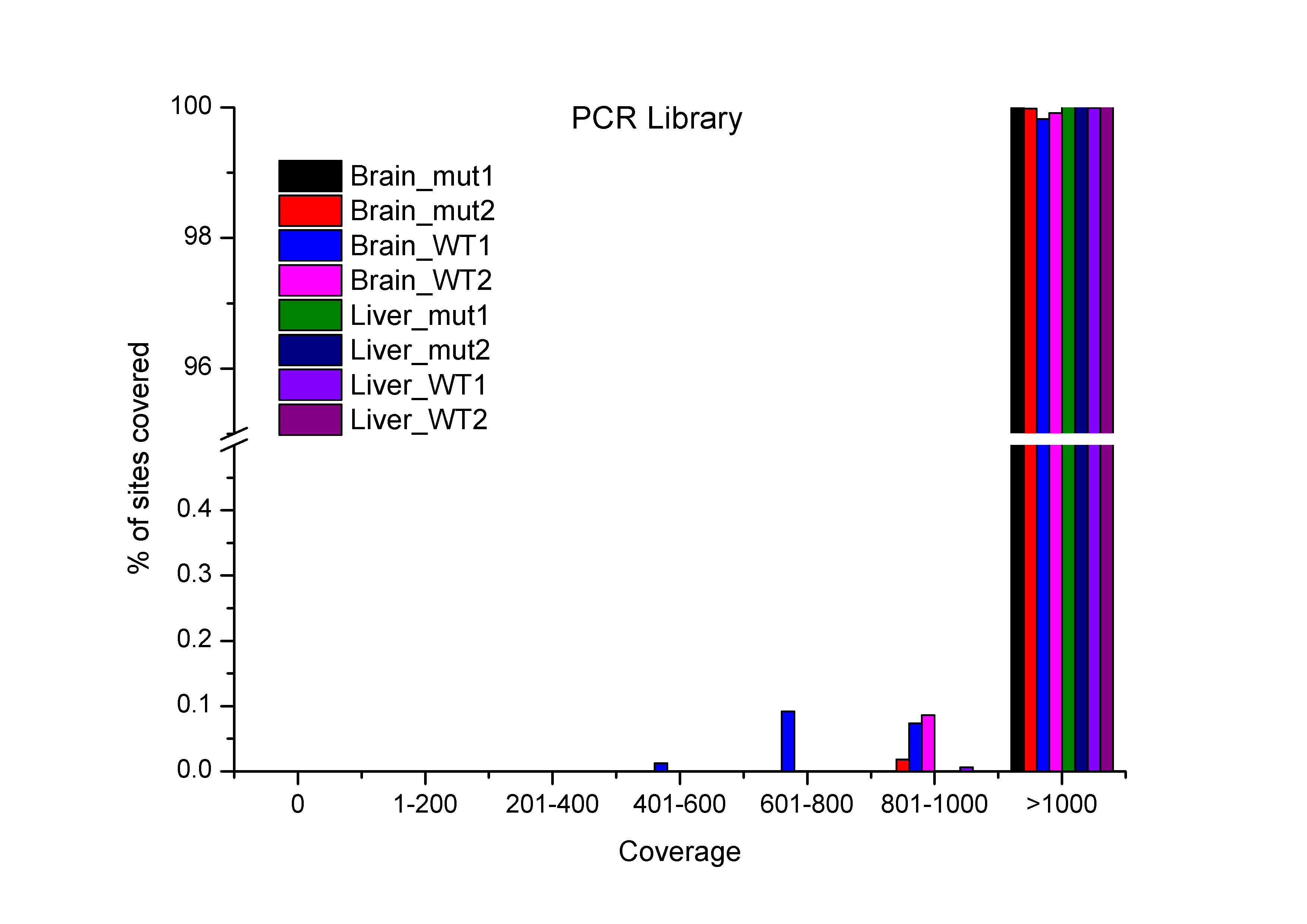


A


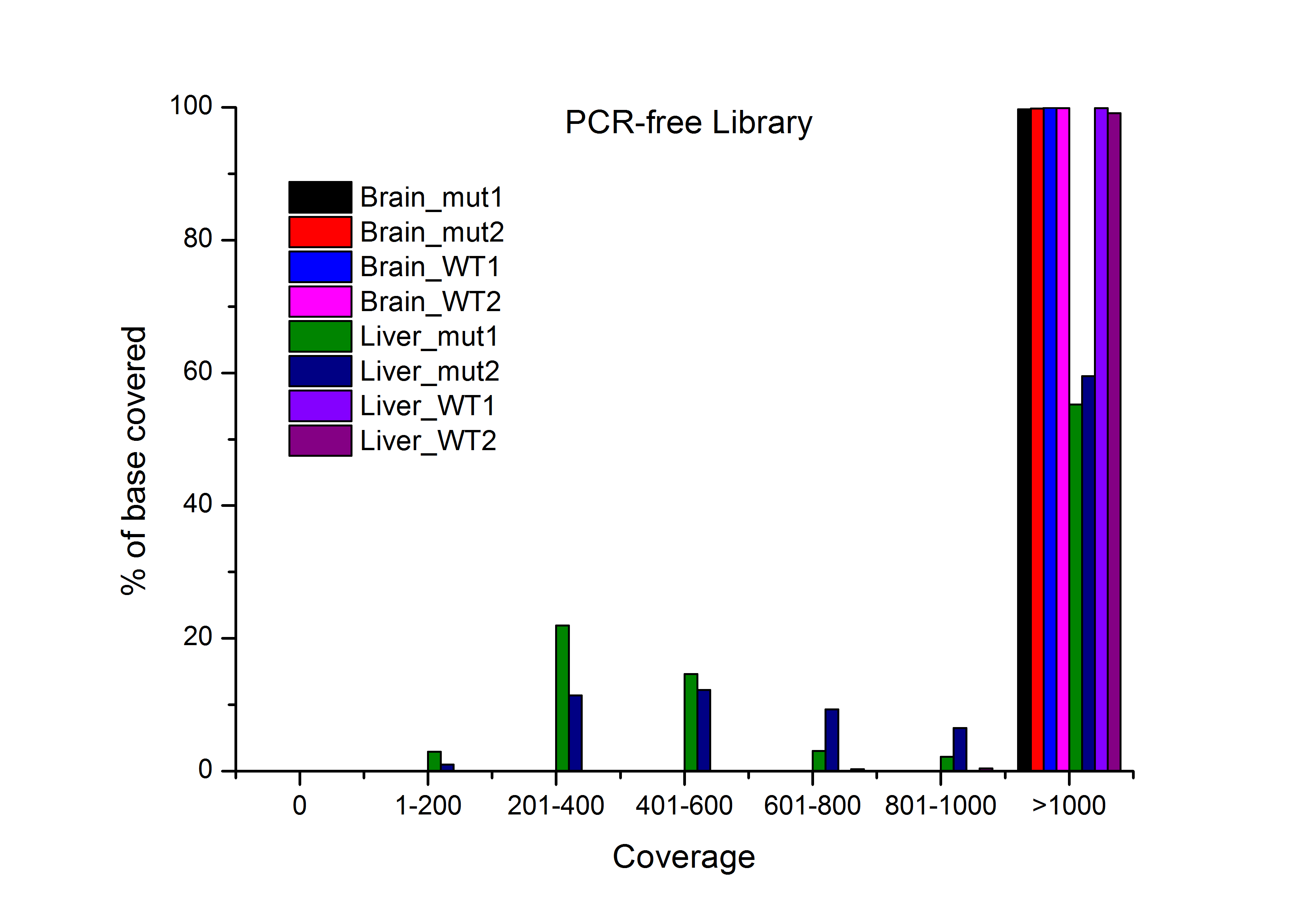


B

Supplementary Figure 5. A summary of base coverage among individual mitoRCA-seq libraries. Low-cycle PCR and PCR-free libraries are shown in (A) and (B), respectively. The x-axis represents the coverage depths, which are grouped in 7 different bins. The y-axis represents the percentage of bases that are covered at a given depth as determined by mitoRCA-seq read counts. Two wild-type mice (WT1 and WT2) and two *Polg* mutant mice (mut1 and mut2) were used. In each mouse, two different tissues, brain and liver, were used to constructed the mitoRCA-seq libraries.


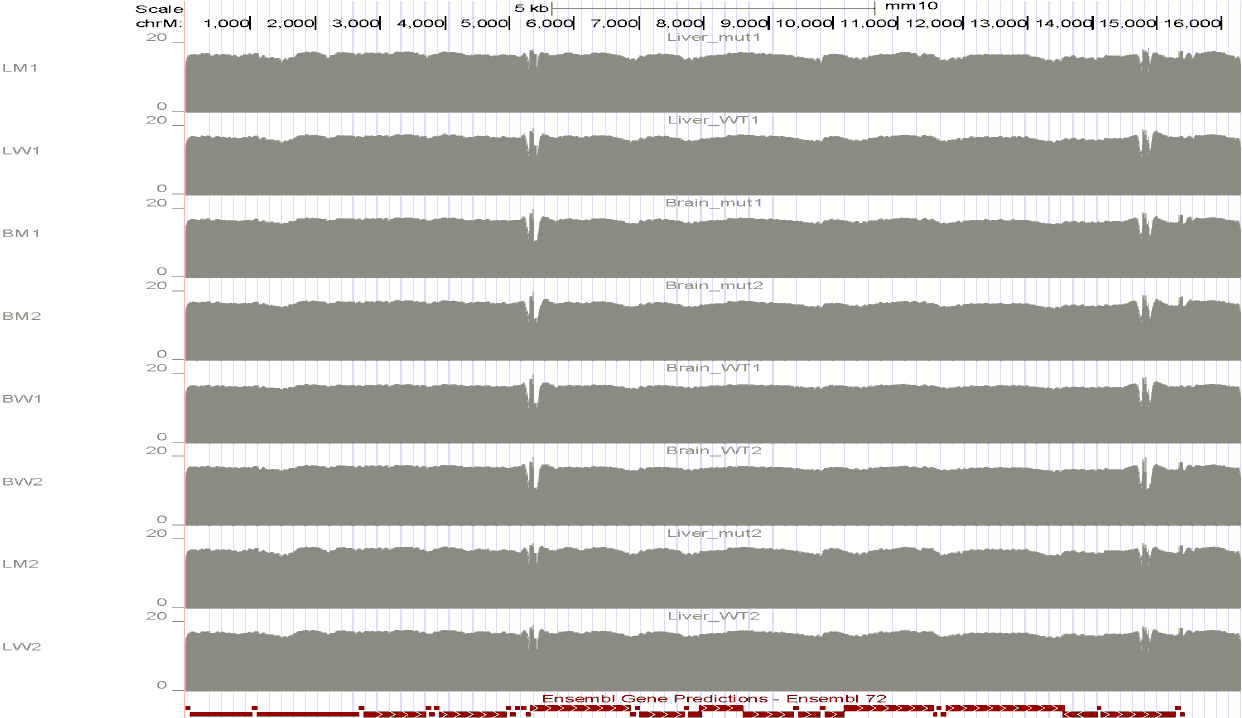


A


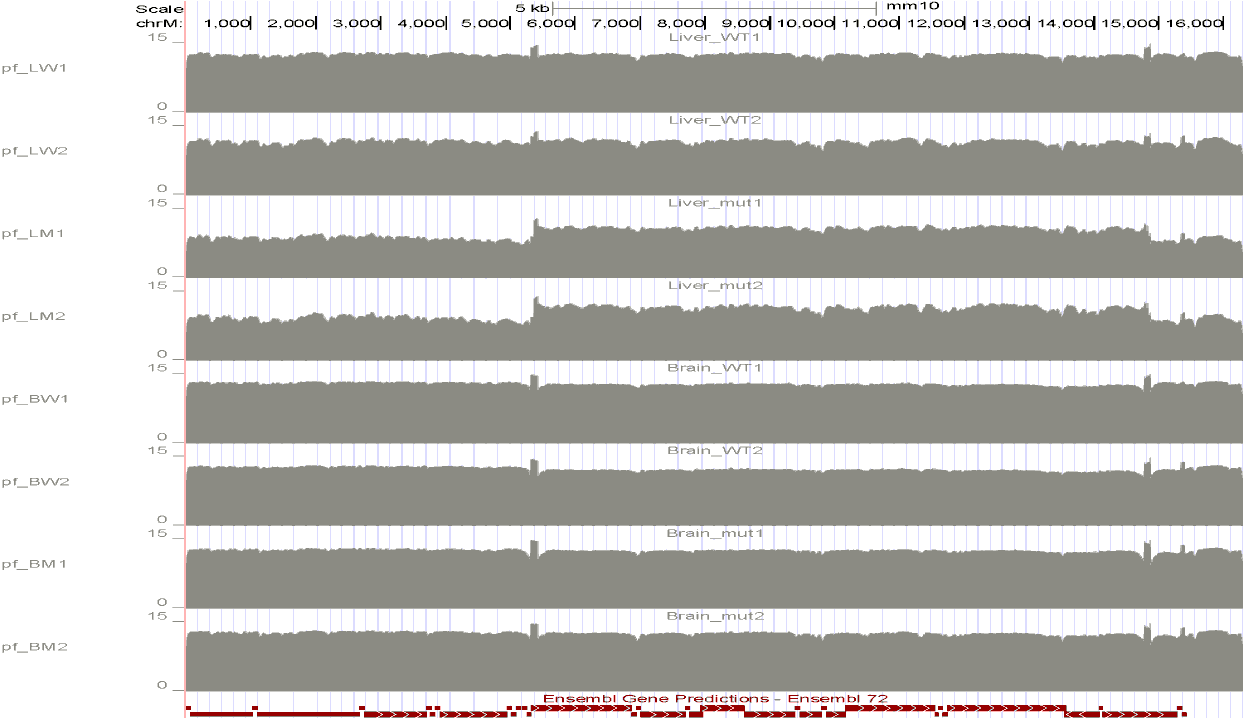


B

Supplementary Figure 6. A graphic view of coverage depth of individual libraries along the entire mitochondrial genome. Low-cycle PCR and PCR-free libraries are shown in (A) and (B), respectively. Liver and brain tissues were obtained from two wild-type (WT1 and WT2) and two Polg mutant mice (mut1 and mut2). The coverage depths of individual mitochondrial bases (x-axis) are log2 transformed and shown on the y-axis. The Ensembl gene annotation is shown at the bottom panel. The mitochondrial genome from mm10 was used for reads alignment.


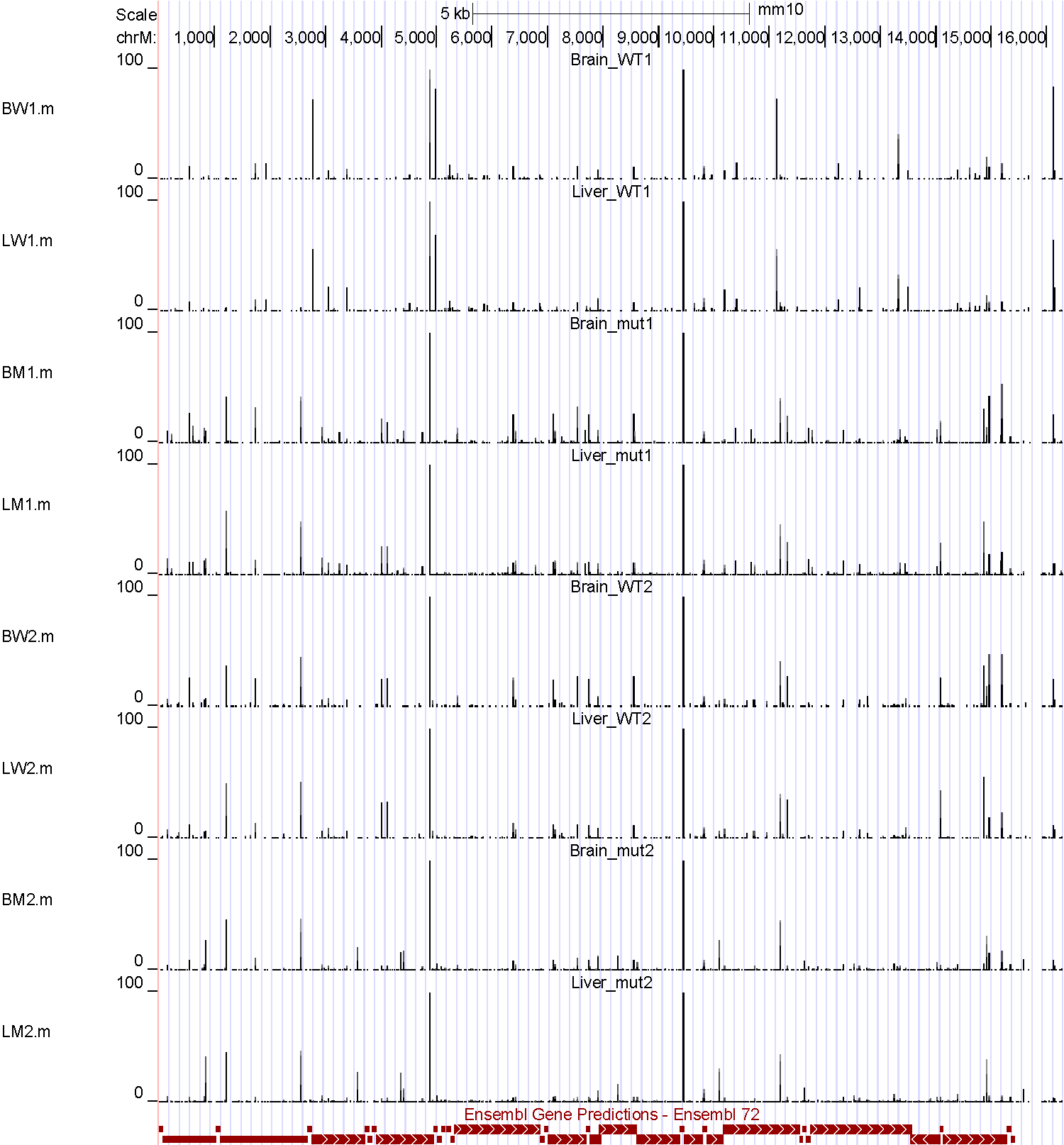


A

B


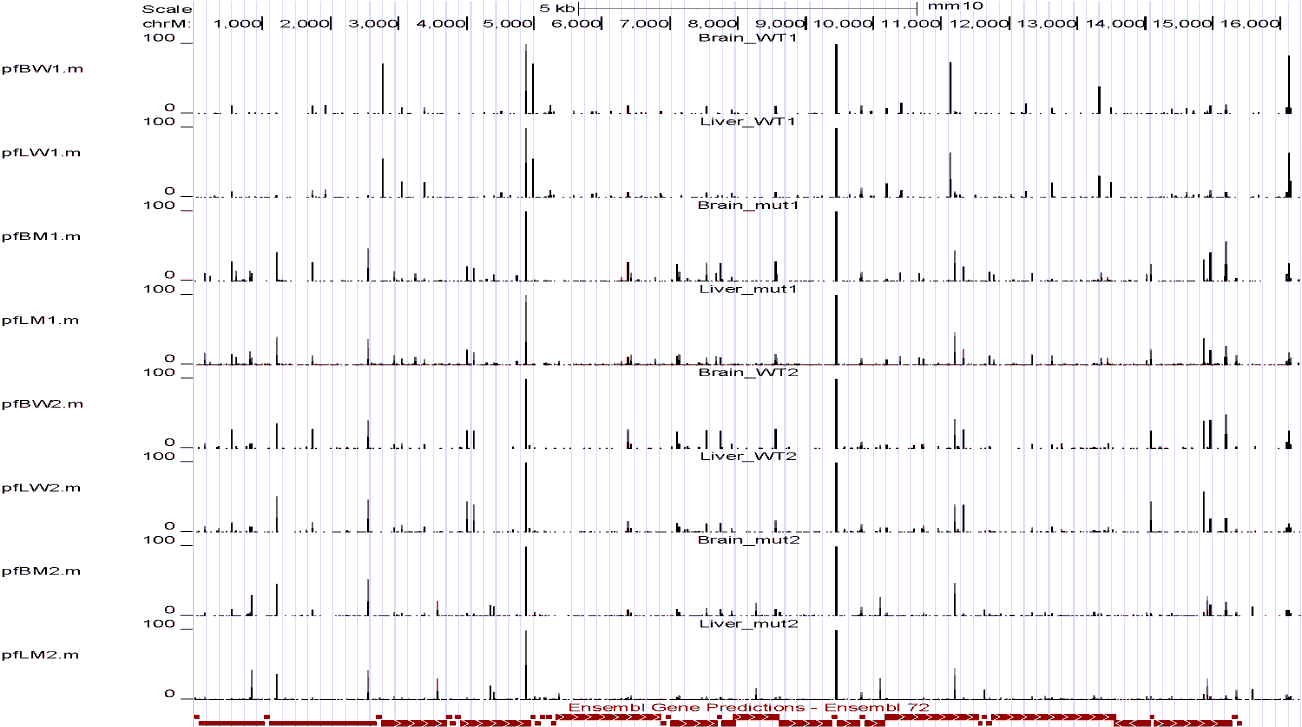


Supplementary Figure 7. A graphic view of the mutation frequencies determined by individual mitoRCA-seq libraries. Low-cycle PCR and PCR-free libraries are shown in (A) and (B), respectively. Liver and brain tissues were obtained from two wild-type (WT1 and WT2) and two Polg mutant mice (mut1 and mut2). The height of the vertical bar (Y-axis) represents the mutation frequency (100 denotes 100%). The Ensembl Gene annotation is shown at the bottom panel. The mitochondrial genome from mm10 was used for reads alignment.

**
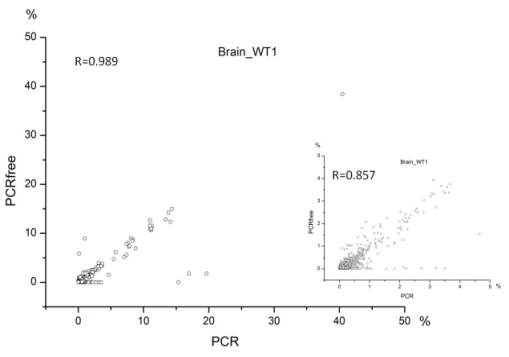

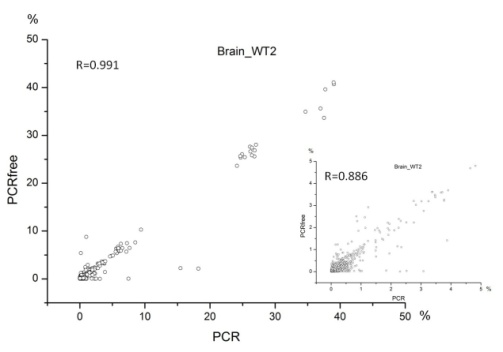
**

**
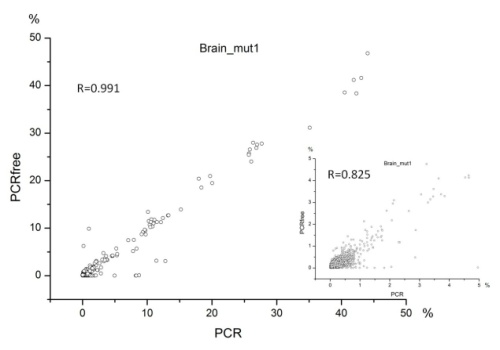

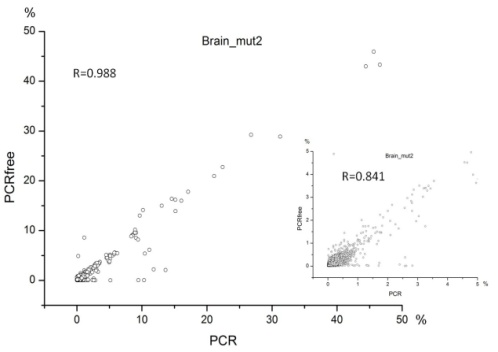
**

**
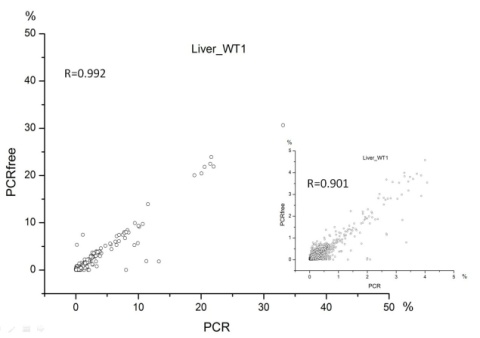

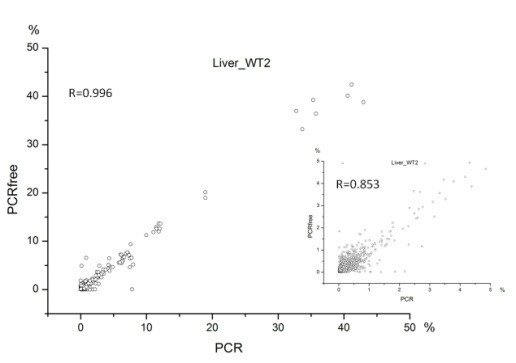
**

**
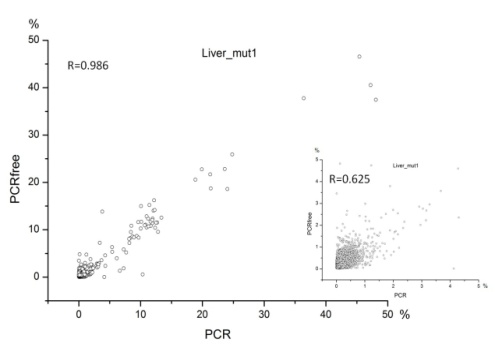

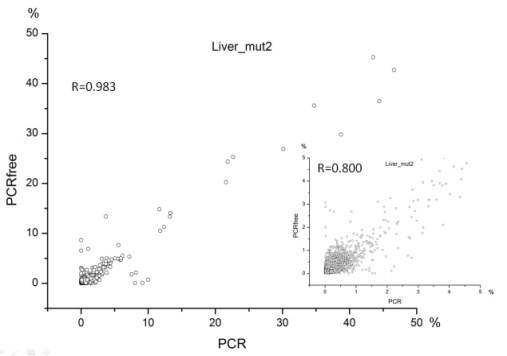
**

Supplementary Figure 8. The SNV frequencies determined by PCR-free and low-cycle PCR procedures are highly correlated. For each tissue (brain or liver) of a given mice (wild-type or *Polg* mutant), its mitochondrial mutation profile was analyzed by mitoRCA-seq either without any PCR step or with a low-cycle PCR procedure. The mutation frequencies resulting from each pair of mitoRCA-seq libraries (low-cycle PCR vs. PCR-free) were used to compute their correlation coefficient, which was either based on all mutation sites (maximum percentage of 50% is shown) or those sites with a mutation frequency less than 5% (the inset in each panel).


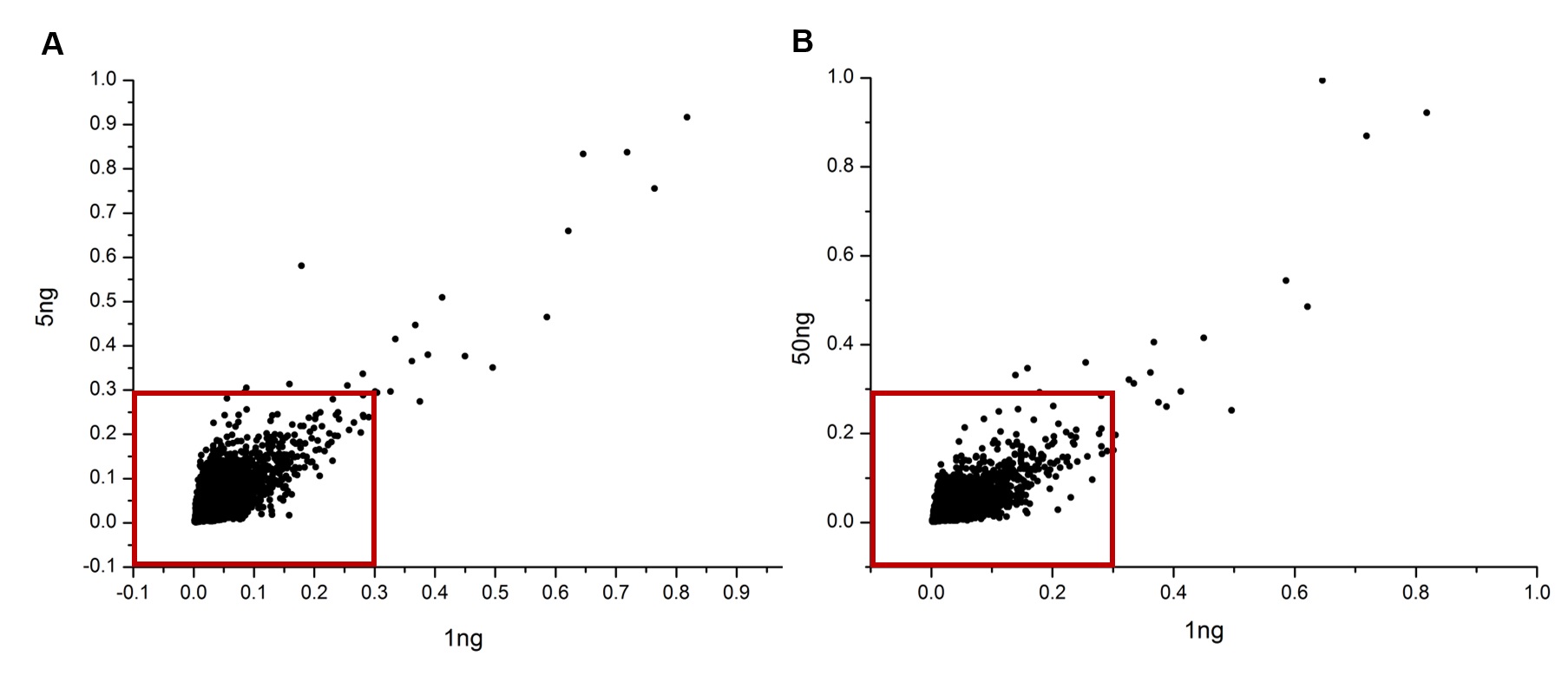


**C**

**R = 0.9621**

**R = 0.9895**


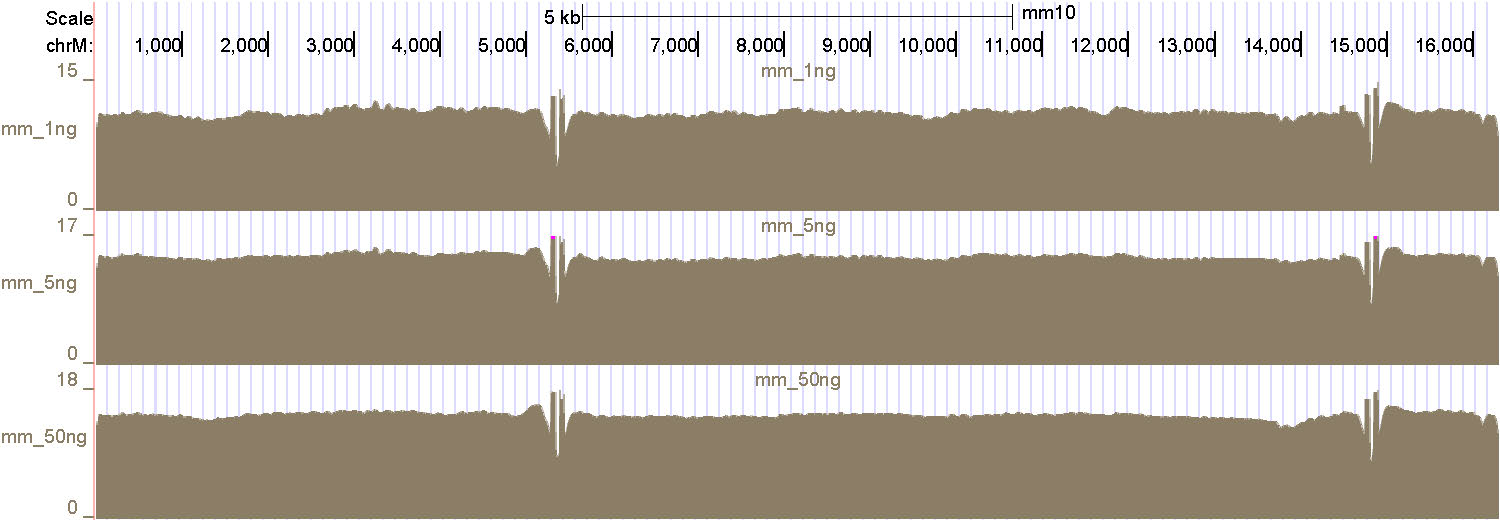


Supplementary Figure 9. The SNV frequencies are highly correlated between low and high input materials. Comparison of SNV frequencies between libraries constructed from 1 ng and 5 ng (A) of mouse total DNA or between 1 ng and 50 ng of total DNA (B). Frequency from 0% to 1% was shown. Panel C shows the coverage of three mitoRCA-seq libraries starting from 1 ng, 5 ng and 50 ng of total DNA, the coverage depths of individual mitochondrial bases (x-axis) are log2 transformed and shown on the y-axis.


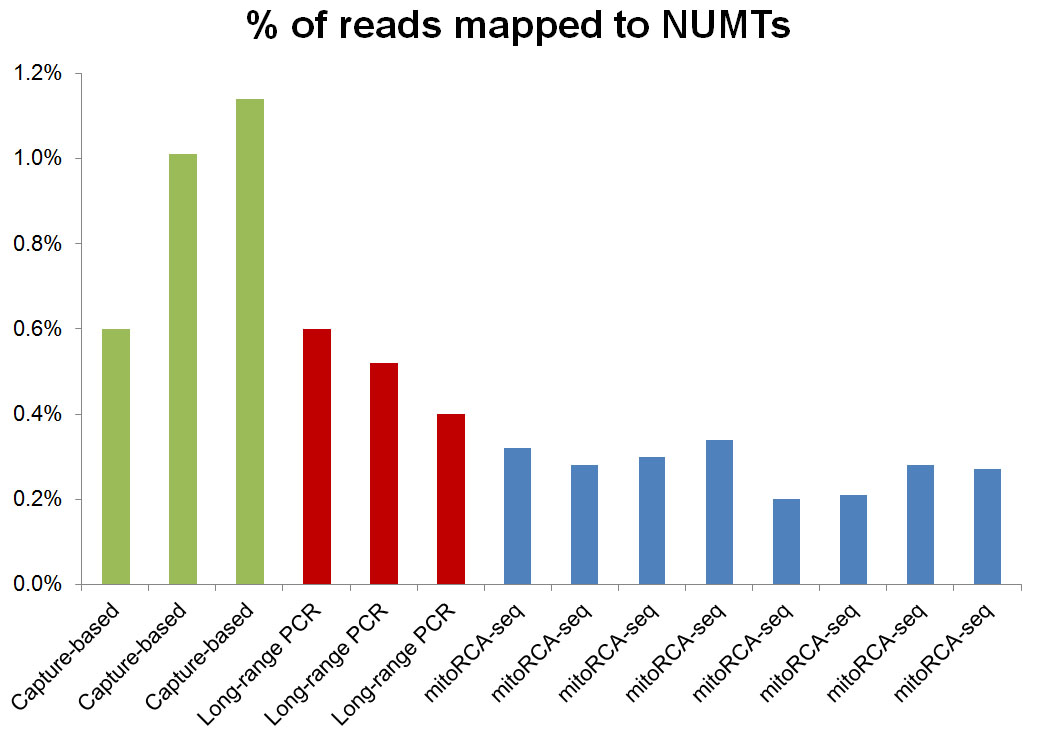


**Capture-based Long-range PCR mitoRCA-seq**

Supplementary Figure 10. Comparison of Numt contaminations among different mitochondrial sequencing methods. The Y-axis represents the percentage of Numt-derived reads in the high-quality mappable reads of each library. In addition to mitoRCA-seq data (this study), the Capture-based and Long-range PCR based mtDNA sequencing data were obtained from Li *et al*.^1^


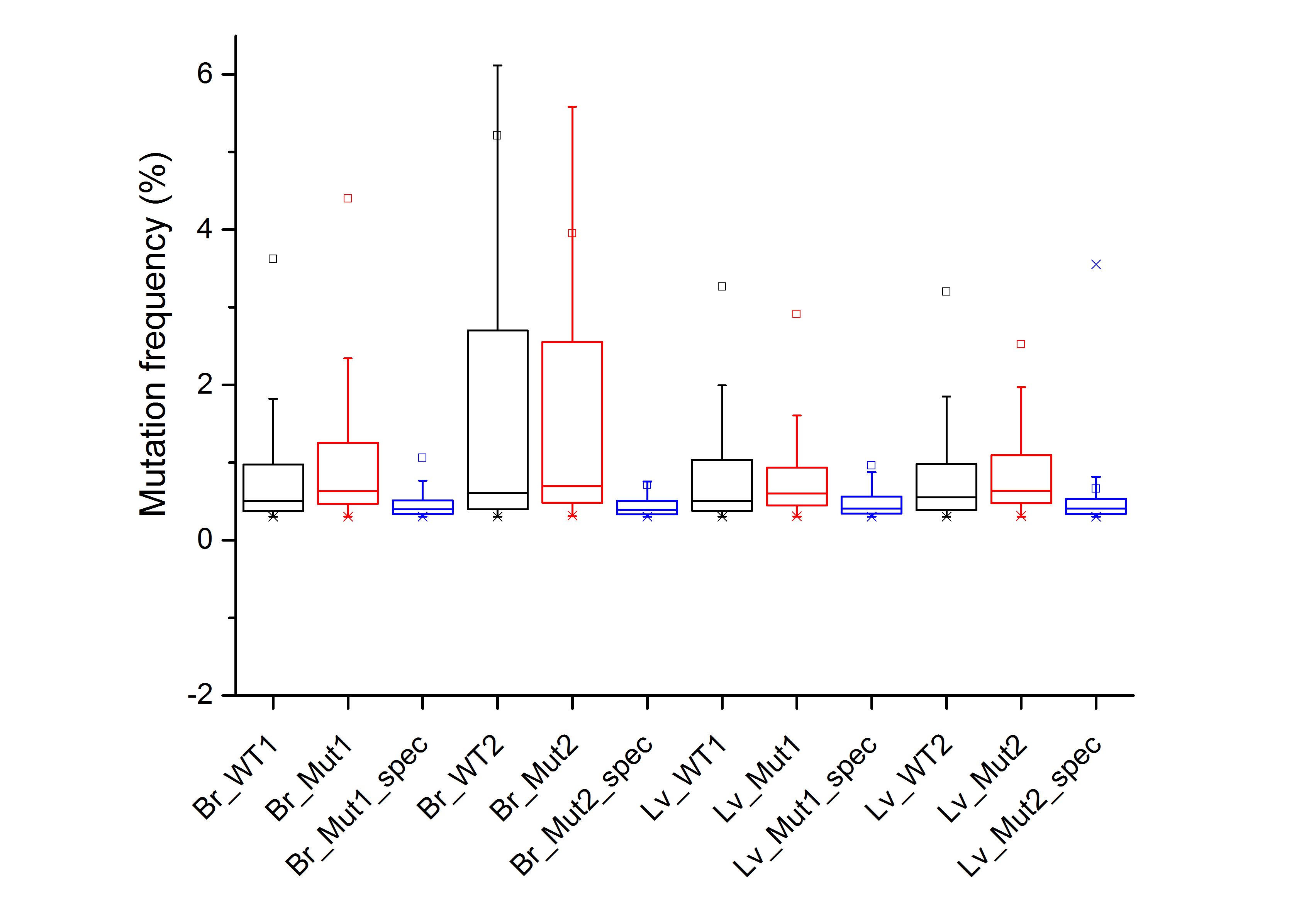


Supplementary Figure 11. Comparison of the mutation frequency between wild-type and *Polg* mutant littermates. For mutation sites identified in a given tissue (Br = brain or Lv = liver) of individual *Polg* mutant and wild-type littermates (e.g. Mut1 vs. WT1), the frequency of these mutation sites in wild-type (black), *Polg* mutant (red) mice and *Polg*-mutant-specific mutation sites (Blue) are shown.


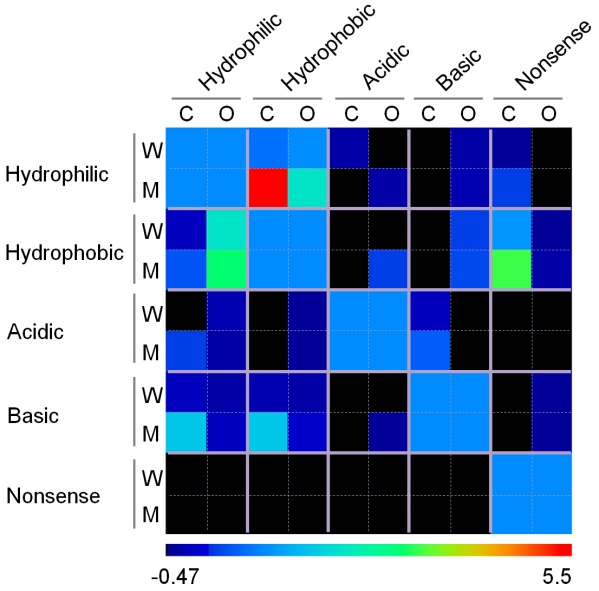

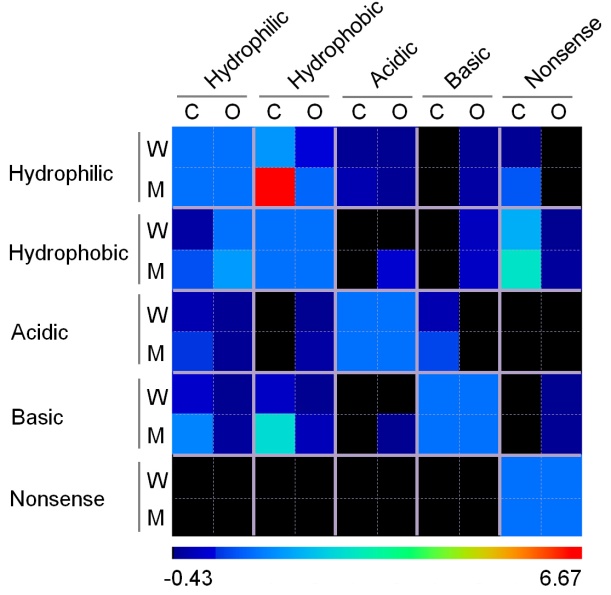


**A**

**B**

Supplementary Figure 12. The effect of mitochondrial mutations on altering amino acid property. Two plots are shown based on the results derived from the brain (A) and liver (B) of the wild-type (WT2) and *Polg* mutant (Mut2) mice. C→T transitions (and its reciprocal mutation G→A) (C) were compared with other types of mutations (O) in their ability to alter amino acid property. For individual mutations identified by mitoRCA-seq, the properties of the corresponding amino acids in the reference genome are shown in rows, and the amino acid properties resulting from individual mutations are shown in column. Enrichment scores (Z-score) were then computed for all possible combinations (e.g. hydrophilic to hydrophobic). For each biological replicate of a given tissue, enrichment analyses were performed separately for wild-type (W) and mutant (M) mice, and the results are shown in a combined heat map.


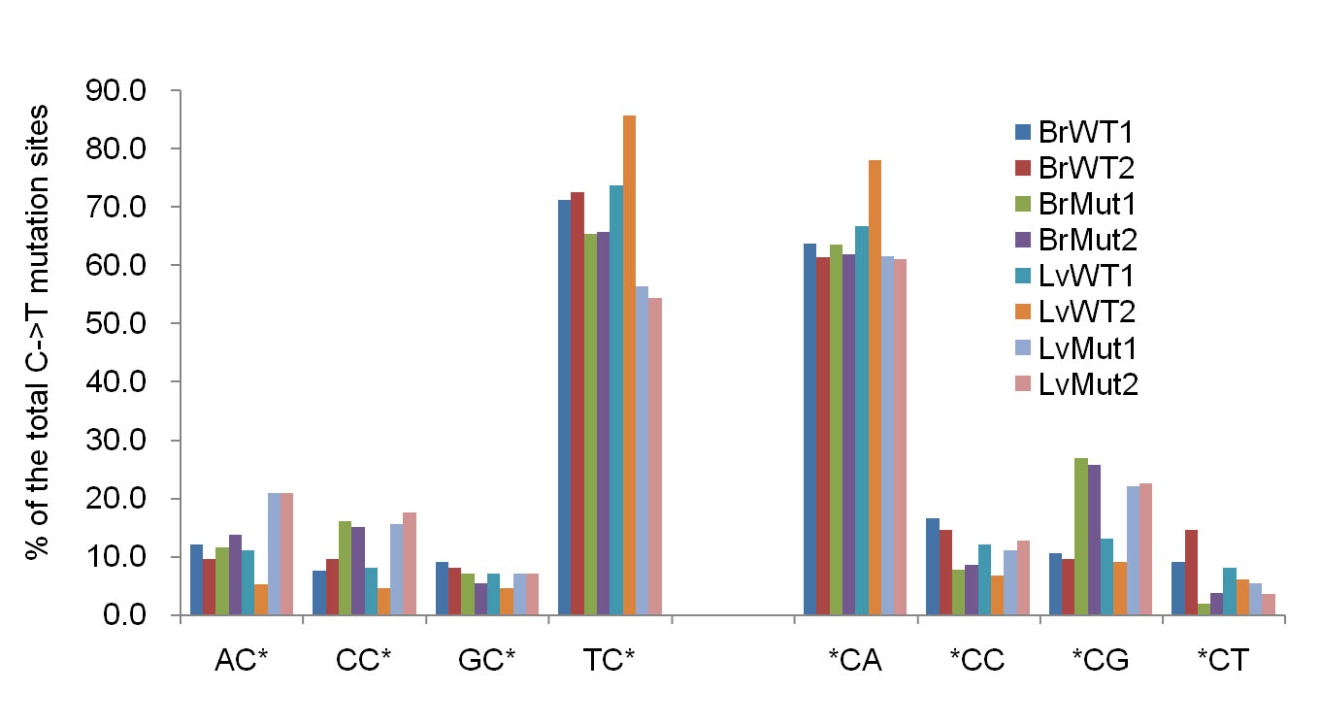


Supplementary Figure 13. The context dependence of the C→T transitions identified in the mouse mitochondrial genome. The frequency of the nucleotide upstream or downstream of the C→T transition sites are plotted. The cytosine next to a * (or any nucleotide) represents the sites that exhibit C→T transition. Eight samples were included in the analysis, including two tissue types (brain = Br and liver = Lv) from two wild-type (WT1 and WT2) and two *Polg* mutant (Mut1 and Mut2) mice.


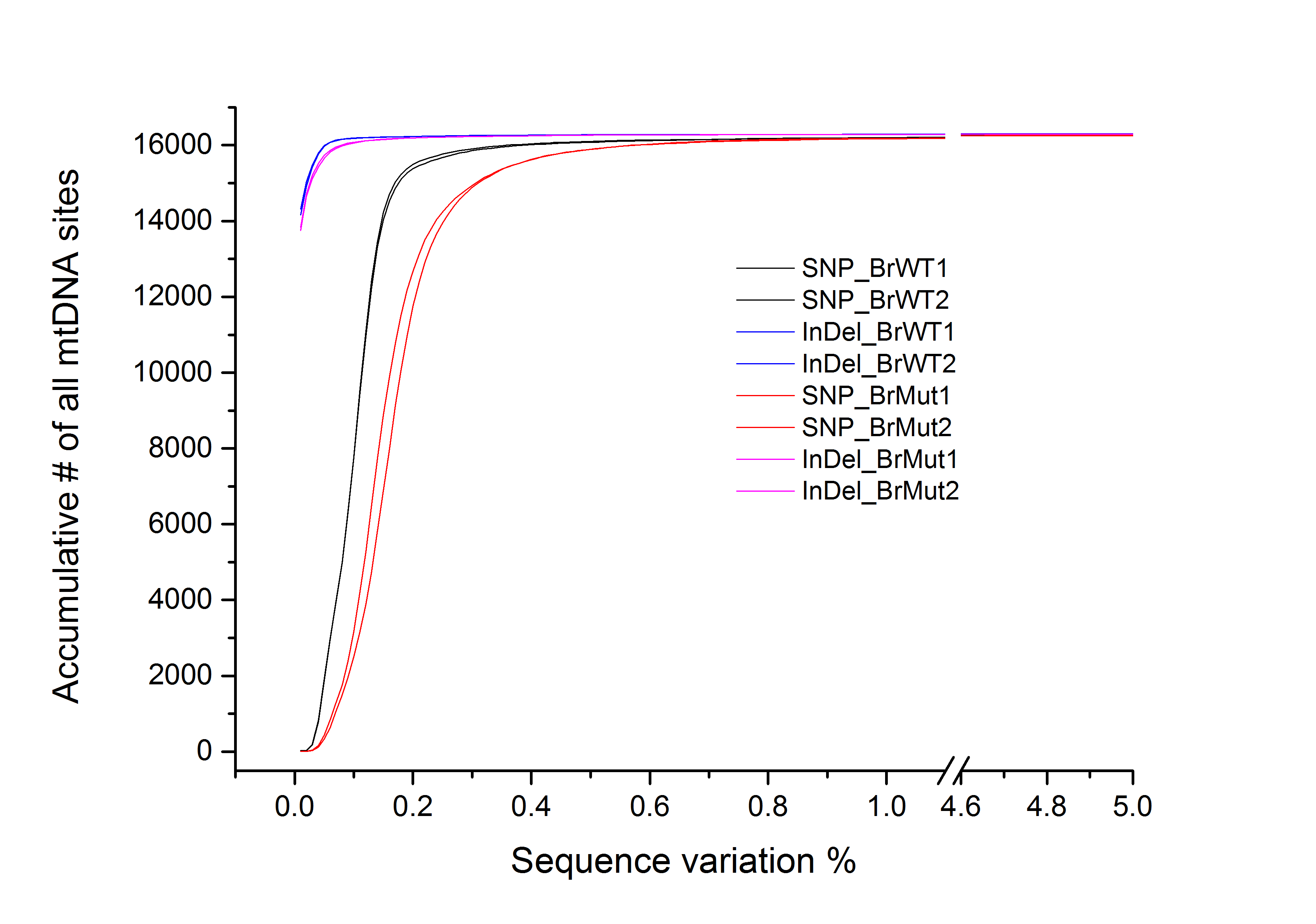


**A**

**B**


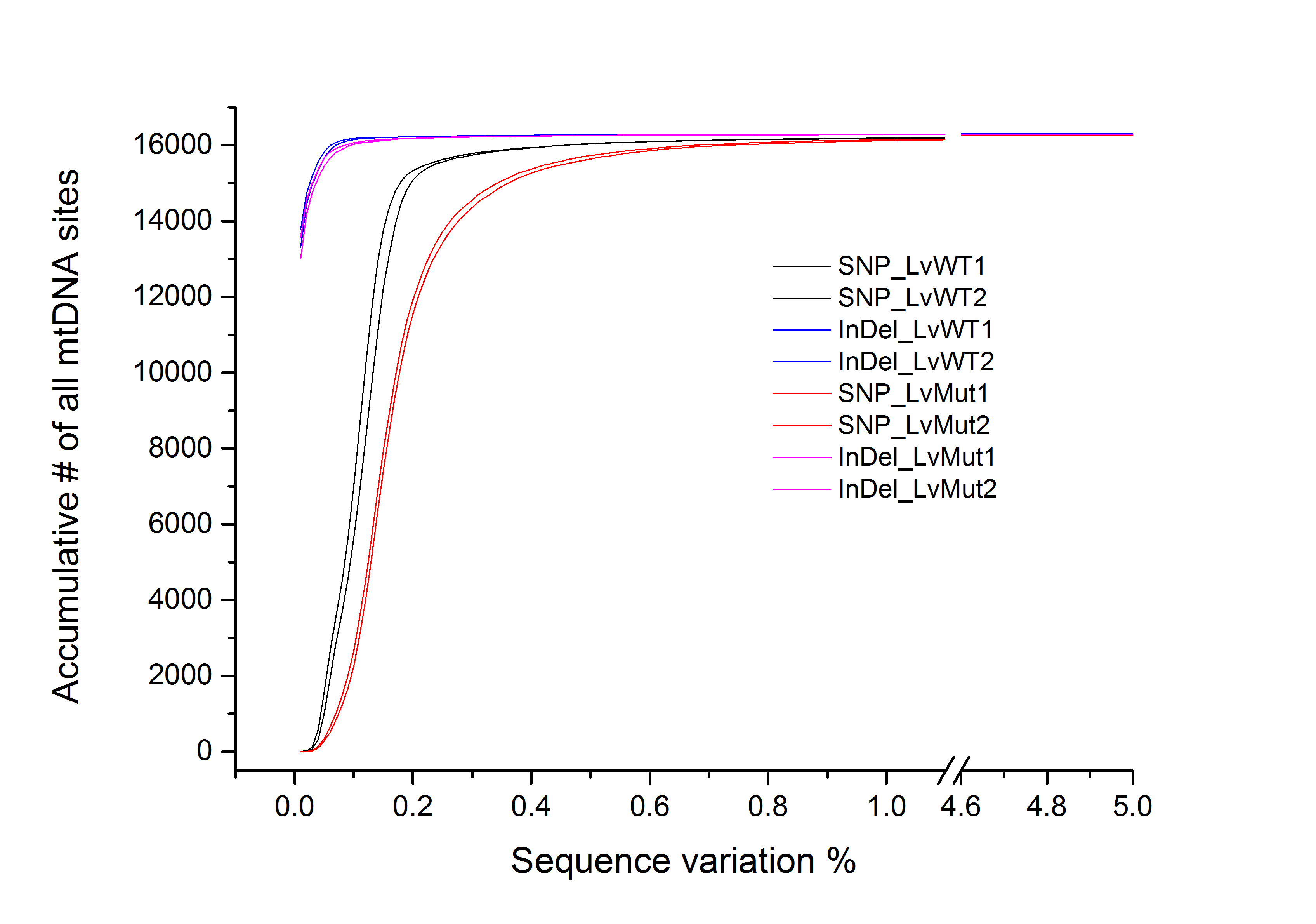


Supplementary Figure 14. Cumulative plots of SNP and small InDel mutations in brain (A) and liver (B). The X-axis represents the frequency of sequence variation (single nucleotide polymorphism or variation, SNV; small insertion/deletions, InDel). Br and Lv is abbreviation of brain and liver, respectively. Four samples are shown for each tissue, consisting of biological replicates derived from two wild-type (WT1 and WT2) and two *Polg* mutant mice (Mut1 and Mut2).

**Supplementary Tables**

**Supplementary Table 1. Primers used for constructing plasmid RCA-seq library.**

| Pr_id | Pr_f pos | Pr_f_seq | Pr_r pos | pr_r_seq |
| --- | --- | --- | --- | --- |
| Pr01 | 38-58 | GACCAATTGCACTACCATCAC | 857-877 | TACGACTGCTTTCCATTCAAG |
| Pr02 | 606-626 | CAGTTCATGTTCTCGGCTATG | 1507-1527 | TGACTAATTCCTCCGAGACCT |
| Pr03 | 1267-1287 | TTGATAACGAGAAAATGCTGG | 2070-2088 | GGCCACGTCAAACACGTACTC |
| Pr04 | 2151-2171 | AACCCTCCCTATCATGAGCTA | 3027-3047 | TTCAGCTTCCCAGTCCTCTAT |
| Pr05 | 2844-2864 | ACCAGAAAAGGAGTGTATGCC | 3647-3667 | ATCCATTCGATTCTCTTACGG |
| Pr06 | 3549-3569 | GAGTACAAGGAGAAGCAACCC | 4413-4433 | TGTAGATAGCAGTGGAATGGC |
| Pr07 | 4413-4433 | GCCATTCCACTGCTATCTACA | 5265-5285 | TGACGACCAACTGTCCATACT |
| Pr08 | 5017-5031 | TTCATGGCACGAAAGTAGTCC | 5981-6001 | TCAGTGGTTATGGCTTTCTGA |
| Pr09 | 5669-5689 | AATTATATCGTCCCGATCAGC | 6618-6638 | TTTCATGTCCATGACGAATCT |
| Pr10 | 6363-6383 | ATTGCCGCAACTAAAAGAAAT | 7257-7277 | GTAAGGTGGTCTCTCACCGAT |
| Pr11 | 7343-7363 | GAAAAGGCTGTTTAAGTTGGG | 8159-8179 | AATGCCTCACTTCTCATGTTG |
| Pr12 | 7905-7925 | ACGCAGGAGAAGAAGAAGAAG | 8841-8861 | TTCTTTAACGGTGTGATCCTG |
| Pr13 | 8680-8700 | CGTACTGCCACCATACTGAAC | 9567-9587 | GAAGTTTCTGACCGTCTTTCC |
| Pr14 | 9316-9336 | ATAAGAGCGACCAAACGAAGT | 10201-10221 | TGGTGAATTTGCAGGTAATGT |
| Pr15 | 10104-10124 | CCGTATAAGGCACTTGTTGAA | 10984-11004 | CGTCGAAGTCTGCTGAATAAG |
| Pr16 | 10776-10796 | TCAGGATTTGAGATGTGGAAA | 11690-11710 | GATGAACTTCAGGGTCAGCTT |
| Pr17 | 11464-11484 | TCAGAGGGGAAATAAAGCATC | 12344-12364 | TCGGAAGTACATCGAGTTTTG |
| Pr18 | 12395-12415 | TACATTAGATCCCCGCTTACC | 13250-13270 | CTCCGATCGTTGTCAGAAGTA |
| Pr19 | 12920-12940 | CAGAAACGCTGGTGAAAGTAA | 13808-13828 | ACTCACGTTAAGGGATTTTGG |
| Pr20 | 13653-13673 | TGAACGAAATAGACAGATCGC | 14483-14503 | CGACAATATGTCCATACGAGC |

Note: Pr refers to Primer, _f and _r refer to the forward and reverse primer respectively. pos denotes position of the primer in the plasmid. Sequence of the plasmid is shown below:

**Full length sequence of plasmid pTEsindbisGFP**

ATTGACGGCGTAGTACACACTATTGAATCAAACAGCCGACCAATTGCACTACCATCACAATGGAGAAGCCAGTAGTAAACGTAGACGTAGACCCCCAGAGTCCGTTTGTCGTGCAACTGCAAAAAAGCTTCCCGCAATTTGAGGTAGTAGCACAGCAGGTCACTCCAAATGACCATGCTAATGCCAGAGCATTTTCGCATCTGGCCAGTAAACTAATCGAGCTGGAGGTTCCTACCACAGCGACGATCTTGGACATAGGCAGCGCACCGGCTCGTAGAATGTTTTCCGAGCACCAGTATCATTGTGTCTGCCCCATGCGTAGTCCAGAAGACCCGGACCGCATGATGAAATACGCCAGTAAACTGGCGGAAAAAGCGTGCAAGATTACAAACAAGAACTTGCATGAGAAGATTAAGGATCTCCGGACCGTACTTGATACGCCGGATGCTGAAACACCATCGCTCTGCTTTCACAACGATGTTACCTGCAACATGCGTGCCGAATATTCCGTCATGCAGGACGTGTATATCAACGCTCCCGGAACTATCTATCATCAGGCTATGAAAGGCGTGCGGACCCTGTACTGGATTGGCTTCGACACCACCCAGTTCATGTTCTCGGCTATGGCAGGTTCGTACCCTGCGTACAACACCAACTGGGCCGACGAGAAAGTCCTTGAAGCGCGTAACATCGGACTTTGCAGCACAAAGCTGAGTGAAGGTAGGACAGGAAAATTGTCGATAATGAGGAAGAAGGAGTTGAAGCCCGGGTCGCGGGTTTATTTCTCCGTAGGATCGACACTTTATCCAGAACACAGAGCCAGCTTGCAGAGCTGGCATCTTCCATCGGTGTTCCACTTGAATGGAAAGCAGTCGTACACTTGCCGCTGTGATACAGTGGTGAGTTGCGAAGGCTACGTAGTGAAGAAAATCACCATCAGTCCCGGGATCACGGGAGAAACCGTGGGATACGCGGTTACACACAATAGCGAGGGCTTCTTGCTATGCAAAGTTACTGACACAGTAAAAGGAGAACGGGTATCGTTCCCTGTGTGCACGTACATCCCGGCCACCATATGCGATCAGATGACTGGTATAATGGCCACGGATATATCACCTGACGATGCACAAAAACTTCTGGTTGGGCTCAACCAGCGAATTGTCATTAACGGTAGGACTAACAGGAACACCAACACCATGCAAAATTACCTTCTGCCGATCATAGCACAAGGGTTCAGCAAATGGGCTAAGGAGCGCAAGGATGATCTTGATAACGAGAAAATGCTGGGTACTAGAGAACGCAAGCTTACGTATGGCTGCTTGTGGGCGTTTCGCACTAAGAAAGTACATTCGTTTTATCGCCCACCTGGAACGCAGACCTGCGTAAAAGTCCCAGCCTCTTTTAGCGCTTTTCCCATGTCGTCCGTATGGACGACCTCTTTGCCCATGTCGCTGAGGCAGAAATTGAAACTGGCATTGCAACCAAAGAAGGAGGAAAAACTGCTGCAGGTCTCGGAGGAATTAGTCATGGAGGCCAAGGCTGCTTTTGAGGATGCTCAGGAGGAAGCCAGAGCGGAGAAGCTCCGAGAAGCACTTCCACCATTAGTGGCAGACAAAGGCATCGAGGCAGCCGCAGAAGTTGTCTGCGAAGTGGAGGGGCTCCAGGCGGACATCGGAGCAGCATTAGTTGAAACCCCGCGCGGTCACGTAAGGATAATACCTCAAGCAAATGACCGTATGATCGGACAGTATATCGTTGTCTCGCCAAACTCTGTGCTGAAGAATGCCAAACTCGCACCAGCGCACCCGCTAGCAGATCAGGTTAAGATCATAACACACTCCGGAAGATCAGGAAGGTACGCGGTCGAACCATACGACGCTAAAGTACTGATGCCAGCAGGAGGTGCCGTACCATGGCCAGAATTCCTAGCACTGAGTGAGAGCGCCACGTTAGTGTACAACGAAAGAGAGTTTGTGAACCGCAAACTATACCACATTGCCATGCATGGCCCCGCCAAGAATACAGAAGAGGAGCAGTACAAGGTTACAAAGGCAGAGCTTGCAGAAACAGAGTACGTGTTTGACGTGGACAAGAAGCGTTGCGTTAAGAAGGAAGAAGCCTCAGGTCTGGTCCTCTCGGGAGAACTGACCAACCCTCCCTATCATGAGCTAGCTCTGGAGGGACTGAAGACCCGACCTGCGGTCCCGTACAAGGTCGAAACAATAGGAGTGATAGGCACACCGGGGTCGGGCAAGTCAGCTATTATCAAGTCAACTGTCACGGCACGAGATCTTGTTACCAGCGGAAAGAAAGAAAATTGTCGCGAAATTGAGGCCGACGTGCTAAGACTGAGGGGTATGCAGATTACGTCGAAGACAGTAGATTCGGTTATGCTCAACGGATGCCACAAAGCCGTAGAAGTGCTGTACGTTGACGAAGCGTTCGCGTGCCACGCAGGAGCACTACTTGCCTTGATTGCTATCGTCAGGCCCCGCAAGAAGGTAGTACTATGCGGAGACCCCATGCAATGCGGATTCTTCAACATGATGCAACTAAAGGTACATTTCAATCACCCTGAAAAAGACATATGCACCAAGACATTCTACAAGTATATCTCCCGGCGTTGCACACAGCCAGTTACAGCTATTGTATCGACACTGCATTACGATGGAAAGATGAAAACCACGAACCCGTGCAAGAAGAACATTGAAATCGATATTACAGGGGCCACAAAGCCGAAGCCAGGGGATATCATCCTGACATGTTTCCGCGGGTGGGTTAAGCAATTGCAAATCGACTATCCCGGACATGAAGTAATGACAGCCGCGGCCTCACAAGGGCTAACCAGAAAAGGAGTGTATGCCGTCCGGCAAAAAGTCAATGAAAACCCACTGTACGCGATCACATCAGAGCATGTGAACGTGTTGCTCACCCGCACTGAGGACAGGCTAGTGTGGAAAACCTTGCAGGGCGACCCATGGATTAAGCAGCCCACTAACATACCTAAAGGAAACTTTCAGGCTACTATAGAGGACTGGGAAGCTGAACACAAGGGAATAATTGCTGCAATAAACAGCCCCACTCCCCGTGCCAATCCGTTCAGCTGCAAGACCAACGTTTGCTGGGCGAAAGCATTGGAACCGATACTAGCCACGGCCGGTATCGTACTTACCGGTTGCCAGTGGAGCGAACTGTTCCCACAGTTTGCGGATGACAAACCACATTCGGCCATTTACGCCTTAGACGTAATTTGCATTAAGTTTTTCGGCATGGACTTGACAAGCGGACTGTTTTCTAAACAGAGCATCCCACTAACGTACCATCCCGCCGATTCAGCGAGGCCGGTAGCTCATTGGGACAACAGCCCAGGAACCCGCAAGTATGGGTACGATCACGCCATTGCCGCCGAACTCTCCCGTAGATTTCCGGTGTTCCAGCTAGCTGGGAAGGGCACACAACTTGATTTGCAGACGGGGAGAACCAGAGTTATCTCTGCACAGCATAACCTGGTCCCGGTGAACCGCAATCTTCCTCACGCCTTAGTCCCCGAGTACAAGGAGAAGCAACCCGGCCCGGTCAAAAAATTCTTGAACCAGTTCAAACACCACTCAGTACTTGTGGTATCAGAGGAAAAAATTGAAGCTCCCCGTAAGAGAATCGAATGGATCGCCCCGATTGGCATAGCCGGTGCAGATAAGAACTACAACCTGGCTTTCGGGTTTCCGCCGCAGGCACGGTACGACCTGGTGTTCATCAACATTGGAACTAAATACAGAAACCACCACTTTCAGCAGTGCGAAGACCATGCGGCGACCTTAAAAACCCTTTCGCGTTCGGCCCTGAATTGCCTTAACCCAGGAGGCACCCTCGTGGTGAAGTCCTATGGCTACGCCGACCGCAACAGTGAGGACGTAGTCACCGCTCTTGCCAGAAAGTTTGTCAGGGTGTCTGCAGCGAGACCAGATTGTGTCTCAAGCAATACAGAAATGTACCTGATTTTCCGACAACTAGACAACAGCCGTACACGGCAATTCACCCCGCACCATCTGAATTGCGTGATTTCGTCCGTGTATGAGGGTACAAGAGATGGAGTTGGAGCCGCGCCGTCATACCGCACCAAAAGGGAGAATATTGCTGACTGTCAAGAGGAAGCAGTTGTCAACGCAGCCAATCCGCTGGGTAGACCAGGCGAAGGAGTCTGCCGTGCCATCTATAAACGTTGGCCGACCAGTTTTACCGATTCAGCCACGGAGACAGGCACCGCAAGAATGACTGTGTGCCTAGGAAAGAAAGTGATCCACGCGGTCGGCCCTGATTTCCGGAAGCACCCAGAAGCAGAAGCCTTGAAATTGCTACAAAACGCCTACCATGCAGTGGCAGACTTAGTAAATGAACATAACATCAAGTCTGTCGCCATTCCACTGCTATCTACAGGCATTTACGCAGCCGGAAAAGACCGCCTTGAAGTATCACTTAACTGCTTGACAACCGCGCTAGACAGAACTGACGCGGACGTAACCATCTATTGCCTGGATAAGAAGTGGAAGGAAAGAATCGACGCGGCACTCCAACTTAAGGAGTCTGTAACAGAGCTGAAGGATGAAGATATGGAGATCGACGATGAGTTAGTATGGATCCATCCAGACAGTTGCTTGAAGGGAAGAAAGGGATTCAGTACTACAAAAGGAAAATTGTATTCGTACTTCGAAGGCACCAAATTCCATCAAGCAGCAAAAGACATGGCGGAGATAAAGGTCCTGTTCCCTAATGACCAGGAAAGTAATGAACAACTGTGTGCCTACATATTGGGTGAGACCATGGAAGCAATCCGCGAAAAGTGCCCGGTCGACCATAACCCGTCGTCTAGCCCGCCCAAAACGTTGCCGTGCCTTTGCATGTATGCCATGACGCCAGAAAGGGTCCACAGACTTAGAAGCAATAACGTCAAAGAAGTTACAGTATGCTCCTCCACCCCCCTTCCTAAGCACAAAATTAAGAATGTTCAGAAGGTTCAGTGCACGAAAGTAGTCCTGTTTAATCCGCACACTCCCGCATTCGTTCCCGCCCGTAAGTACATAGAAGTGCCAGAACAGCCTACCGCTCCTCCTGCACAGGCCGAGGAGGCCCCCGAAGTTGTAGCGACACCGTCACCATCTACAGCTGATAACACCTCGCTTGATGTCACAGACATCTCACTGGATATGGATGACAGTAGCGAAGGCTCACTTTTTTCGAGCTTTAGCGGATCGGACAACTCTATTACTAGTATGGACAGTTGGTCGTCAGGACCTAGTTCACTAGAGATAGTAGACCGAAGGCAGGTGGTGGTGGCTGACGTTCATGCCGTCCAAGAGCCTGCCCCTATTCCACCGCCAAGGCTAAAGAAGATGGCCCGCCTGGCAGCGGCAAGAAAAGAGCCCACTCCACCGGCAAGCAATAGCTCTGAGTCCCTCCACCTCTCTTTTGGTGGGGTATCCATGTCCCTCGGATCAATTTTCGACGGAGAGACGGCCCGCCAGGCAGCGGTACAACCCCTGGCAACAGGCCCCACGGATGTGCCTATGTCTTTCGGATCGTTTTCCGACGGAGAGATTGATGAGCTGAGCCGCAGAGTAACTGAGTCCGAACCCGTCCTGTTTGGATCATTTGAACCGGGCGAAGTGAACTCAATTATATCGTCCCGATCAGCCGTATCTTTTCCACTACGCAAGCAGAGACGTAGACGCAGGAGCAGGAGGACTGAATACTGACTAACCGGGGTAGGTGGGTACATATTTTCGACGGACACAGGCCCTGGGCACTTGCAAAAGAAGTCCGTTCTGCAGAACCAGCTTACAGAACCGACCTTGGAGCGCAATGTCCTGGAAAGAATTCATGCCCCGGTGCTCGACACGTCGAAAGAGGAACAACTCAAACTCAGGTACCAGATGATGCCCACCGAAGCCAACAAAAGTAGGTACCAGTCTCGTAAAGTAGAAAATCAGAAAGCCATAACCACTGAGCGACTACTGTCAGGACTACGACTGTATAACTCTGCCACAGATCAGCCAGAATGCTATAAGATCACCTATCCGAAACCATTGTACTCCAGTAGCGTACCGGCGAACTACTCCGATCCACAGTTCGCTGTAGCTGTCTGTAACAACTATCTGCATGAGAACTATCCGACAGTAGCATCTTATCAGATTACTGACGAGTACGATGCTTACTTGGATATGGTAGACGGGACAGTCGCCTGCCTGGATACTGCAACCTTCTGCCCCGCTAAGCTTAGAAGTTACCCGAAAAAACATGAGTATAGAGCCCCGAATATCCGCAGTGCGGTTCCATCAGCGATGCAGAACACGCTACAAAATGTGCTCATTGCCGCAACTAAAAGAAATTGCAACGTCACGCAGATGCGTGAACTGCCAACACTGGACTCAGCGACATTCAATGTCGAATGCTTTCGAAAATATGCATGTAATGACGAGTATTGGGAGGAGTTCGCTCGGAAGCCAATTAGGATTACCACTGAGTTTGTCACCGCATATGTAGCTAGACTGAAAGGCCCTAAGGCCGCCGCACTATTTGCAAAGACGTATAATTTGGTCCCATTGCAAGAAGTGCCTATGGATAGATTCGTCATGGACATGAAAAGAGACGTGAAAGTTACACCAGGCACGAAACACACAGAAGAAAGACCGAAAGTACAAGTGATACAAGCCGCAGAACCCCTGGCGACTGCTTACTTATGCGGGATTCACCGGGAATTAGTGCGTAGGCTTACGGCCGTCTTGCTTCCAAACATTCACACGCTTTTTGACATGTCGGCGGAGGATTTTGATGCAATCATAGCAGAACACTTCAAGCAAGGCGACCCGGTACTGGAGACGGATATCGCATCATTCGACAAAAGCCAAGACGACGCTATGGCGTTAACCGGTCTGATGATCTTGGAGGACCTGGGTGTGGATCAACCACTACTCGACTTGATCGAGTGCGCCTTTGGAGAAATATCATCCACCCATCTACCTACGGGTACTCGTTTTAAATTCGGGGCGATGATGAAATCCGGAATGTTCCTCACACTTTTTGTCAACACAGTTTTGAATGTCGTTATCGCCAGCAGAGTACTAGAAGAGCGGCTTAAAACGTCCAGATGTGCAGCGTTCATTGGCGACGACAACATCATACATGGAGTAGTATCTGACAAAGAAATGGCTGAGAGGTGCGCCACCTGGCTCAACATGGAGGTTAAGATCATCGACGCAGTCATCGGTGAGAGACCACCTTACTTCTGCGGCGGATTTATCTTGCAAGATTCGGTTACTTCCACAGCGTGCCGCGTGGCGGATCCCCTGAAAAGGCTGTTTAAGTTGGGTAAACCGCTCCCAGCCGACGACGAGCAAGACGAAGACAGAAGACGCGCTCTGCTAGATGAAACAAAGGCGTGGTTTAGAGTAGGTATAACAGGCACTTTAGCAGTGGCCGTGACGACCCGGTATGAGGTAGACAATATTACACCTGTCCTACTGGCATTGAGAACTTTTGCCCAGAGCAAAAGAGCATTCCAAGCCATCAGAGGGGAAATAAAGCATCTCTACGGTGGTCCTAAATAGTCAGCATAGTACATTTCATCTGACTAATACTACAACACCACCACCATGAATAGAGGATTCTTTAACATGCTCGGCCGCCGCCCCTTCCCGGCCCCCACTGCCATGTGGAGGCCGCGGAGAAGGAGGCAGGCGGCCCCGATGCCTGCCCGCAACGGGCTGGCTTCTCAAATCCAGCAACTGACCACAGCCGTCAGTGCCCTAGTCATTGGACAGGCAACTAGACCTCAACCCCCACGTCCACGCCCGCCACCGCGCCAGAAGAAGCAGGCGCCCAAGCAACCACCGAAGCCGAAGAAACCAAAAACGCAGGAGAAGAAGAAGAAGCAACCTGCAAAACCCAAACCCGGAAAGAGACAGCGCATGGCACTTAAGTTGGAGGCCGACAGATTGTTCGACGTCAAGAACGAGGACGGAGATGTCATCGGGCACGCACTGGCCATGGAAGGAAAGGTAATGAAACCTCTGCACGTGAAAGGAACCATCGACCACCCTGTGCTATCAAAGCTCAAATTTACCAAGTCGTCAGCATACGACATGGAGTTCGCACAGTTGCCAGTCAACATGAGAAGTGAGGCATTCACCTACACCAGTGAACACCCCGAAGGATTCTATAACTGGCACCACGGAGCGGTGCAGTATAGTGGAGGTAGATTTACCATCCCTCGCGGAGTAGGAGGCAGAGGAGACAGCGGTCGTCCGATCATGGATAACTCCGGTCGGGTTGTCGCGATAGTCCTCGGTGGCGCTGATGAAGGAACACGAACTGCCCTTTCGGTCGTCACCTGGAATAGTAAAGGGAAGACAATTAAGACGACCCCGGAAGGGACAGAAGAGTGGTCCGCAGCACCACTGGTCACGGCAATGTGTTTGCTCGGAAATGTGAGCTTCCCATGCGACCGCCCGCCCACATGCTATACCCGCGAACCTTCCAGAGCCCTCGACATCCTTGAAGAGAACGTGAACCATGAGGCCTACGATACCCTGCTCAATGCCATATTGCGGTGCGGATCGTCTGGCAGAAGCAAAAGAAGCGTCATcGACGACTTTACCCTGACCAGCCCCTACTTGGGCACATGCTCGTACTGCCACCATACTGaACCGTGCTTCAGCCCTGTTAAGATCGAGCAGGTCTGGGACGAAGCGGACGATAACACCATACGCATACAGACTTCCGCCCAGTTTGGATACGACCAtAGCGGAGCAGCAAGCGCAAACAAGTACCGCTACATGTCGCTTAAGCAGGATCACACCGTTAAAGAAGGCACCATGGATGACATCAAGATTAGCACCTCAGGACCGTGTAGAAGGCTTAGCTACAAAGGATACTTTCTCCTCGCAAAATGCCCTCCAGGGGACAGCGTAACGGTTAGCATAGTGAGTAGCAACTCAGCAACGTCATGTACACTGGCCCGCAAGATAAAACCAAAATTCGTGGGACGGGAAAAATATGATCTACCTCCCGTTCACGGTAAAAAAATTCCTTGCACAGTGTACGACCGTCTGAAAGAAACAACTGCAGGCTACATCACTATGCACAGGCCGgGACCGCACGCTTATACATCCTACCTGGAAGAATCATCAGGGAAAGTTTACGCAAAGCCGCCATCTGGGAAGAACATTACGTATGAGTGCAAGTGCGGCGACTACAAGACCGGAACCGTTTCGACCCGCACCGAAATCACTGGTTGCACCGCCATCAAGCAGTGCGTCGCCTATAAGAGCGACCAAACGAAGTGGGTCTTCAACTCACCGGACTTGATCAGACATGACGACCACACGGCCCAAGGGAAATTGCATTTGCCTTTCAAGTTGATCCCGAGTACCTGCATGGTCCCTGTTGCCCACGCGCCGAATGTAATACATGGCTTTAAACACATCAGCCTCCAATTAGATACAGACCACTTGACATTGCTCACCACCAGGAGACTAGGGGCAAACCCGGAACCAACCACTGAATGGATCGTCGGAAAGACGGTCAGAAACTTCACCGTCGACCGAGATGGCCTGGAATACATATGGGGAAATCATGAGCCAGTGAGGGTCTATGCCCAAGAGTCAGCACCAGGAGACCCTCACGGATGGCCACACGAAATAGTACAGCATTACTACCATCGCCATCCTGTGTACACCATCTTAGCCGTCGCATCAGCTACCGTGGCGATGATGATTGGCGTAACTGTTGCAGTGTTATGTGCCTGTAAAGCGCGCCGTGAGTGCCTGACGCCATACGCCCTGGCCCCAAACGCCGTAATCCCAACTTCGCTGGCACTCTTGTGCTGCGTTAGGTCGGCCAATGCTGAAACGTTCACCGAGACCATGAGTTACTTGTGGTCGAACAGTCAGCCGTTCTTCTGGGTCCAGTTGTGCATACCTTTGGCCGCTTTCATCGTTCTAATGCGCTGCTGCTCCTGCTGCCTGCCTTTTTTAGTGGTTGCCGGCGCCTACCTGGCGAAGGTAGACGCCTACGAACATGCGACCACTGTTCCAAATGTGCCACAGATACCGTATAAGGCACTTGTTGAAAGGGCAGGGTATGCCCCGCTCAATTTGGAGATCACTGTCATGTCCTCGGAGGTTTTGCCTTCCACCAACCAAGAGTACATTACCTGCAAATTCACCACTGTGGTCCCCTCCCCAAAAATCAAATGCTGCGGCTCCTTGGAATGTCAGCCGGCCGCTCATGCAGACTATACCTGCAAGGTCTTCGGAGGGGTCTACCCCTTTATGTGGGGAGGAGCGCAATGTTTTTGCGACAGTGAGAACAGCCAGATGAGTGAGGCGTACGTCGAATTGTCAGCAGATTGCGCGTCTGACCACGCGCAGGCGATTAAGGTGCACACTGCCGCGATGAAAGTAGGACTGCGTATTGTGTACGGGAACACTACCAGTTTCCTAGATGTGTACGTGAACGGAGTCACACCAGGAACGTCTAAAGACTTGAAAGTCATAGCTGGACCAATTTCAGCATCaTTTACGCCATTCGATCATAAGGTCGTTATCCATCGCGGCCTGGTGTACAACTATGACTTCCCGGAATATGGAGCGATGAAACCAGGAGCGTTTGGAGACATTCAAGCTACCTCCTTGACTAGCAAGGATCTCATCGCCAGCACAGACATTAGGCTACTCAAGCCTTCCGCCAAGAAtGTGCATGTCCCGTACACGCAGGCCgCATCAGGATTTGAGATGTGGAAAAACAACTCAGGCCGCCCAtTGCAGGAAACCGCACCTTTCGGGTGTAAGATTGCAGTAAATCCGCTCCGAGCGGTGGACTGTTCATACGGGAACATTCCCATTTCTATTGACATCCCGAACGCTGCCTTTATCAGGACATCAGATGCACCACTGGTCTCAACAGTCAAATGTGAAGTCAGTGAGTGCACTTATTCAGCAGACTTCGaCGGGATGGCCACCCTGCAGTATGTATCCGACCGCGAAGGTCAATGCCCCGTACATTCGCATTCGAGCACAGCAACTCTCCAAGAGTCGACAGTACATGTCCTGGAGAAAGGAGCGGTGACAGTACACTTTAGCACCGCGAGTCCACAGGCGAACTTTATCGTATCGCTGTGTGGGAAGAAGACAACATGCAATGCAGAATGTAAACCACCAGCTGACCATATCGTGAGCACCCCGCACAAAAATGACCAAGAATTTCAAGCCGCCATCTCAAAAACATCATGGAGTTGGCTGTTTGCCCTTTTCGGCGGCGCCTCGTCGCTATTAATTATAGGACTTATGATTTTTGCTTGCAGCATGATGCTGACTAGCACACGAAGATGACgggcccAGGTAGACAATATTACACCTGTCCTACTGGCATTGAGAACTTTTGCCCAGAGCAAAAGAGCATTCCAAGCCATCAGAGGGGAAATAAAGCATCTCTACGGTGGTCCTAAATAGTCAGCATAGTACATTTCATCTGACTAATACTACAACACCACCACCtctagagaatcgccaccatggtgagcaagggcgaggagctgttcaccggggtggtgcccatcctggtcgagctggacggcgacgtaaacggccacaagttcagcgtgtccggcgagggcgagggcgatgccacctacggcaagctgaccctgaagttcatctgcaccaccggcaagctgcccgtgccctggcccaccctcgtgaccaccctgacctacggcgtgcagtgcttcagccgctaccccgaccacatgaagcagcacgacttcttcaagtccgccatgcccgaaggctacgtccaggagcgcaccatcttcttcaaggacgacggcaactacaagacccgcgccgaggtgaagttcgagggcgacaccctggtgaaccgcatcgagctgaagggcatcgacttcaaggaggacggcaacatcctggggcacaagctggagtacaactacaacagccacaacgtctatatcatggccgacaagcagaagaacggcatcaaggtgaacttcaagatccgccacaacatcgaggacggcagcgtgcagctcgccgaccactaccagcagaacacccccatcggcgacggccccgtgctgctgcccgacaaccactacctgagcacccagtccgccctgagcaaagaccccaacgagaagcgcgatcacatggtcctgctggagttcgtgaccgccgccgggatcactctcggcatggacgagctgtacaagtaaagcggccgcgactctagaccatggatcctagaCGCTACGCCCCAATGATCCGACCAGCAAAACTCGATGTACTTCCGAGGAACTGATGTGCATAATGCATCAGGCTGGTACATTAGATCCCCGCTTACCGCGGGCAATATAGCAACACTAAAAACTCGATGTACTTCCGAGGAAGCGCAGTACATAATGCTGCGCAGTGTTGCCACATAACCACTATATTAACCATTTATCTAGCGGACGCCAAAAACTCAATGTATTTCTGAGGAAGCGTGGTGCATAATGCCACGCAGCGTCTGCATAACTTTTATTATTTCTTTTATTAATCAACAAAATTTTGTTTTTAACATTTCAAAAAAAAAAAAAAAAAAAAAAAAAAAAAAAAAAAAAGGGAATTCctcGAGGGGAATTAATTCTTGAAGACGAAAGGGCCAGGTGGCACTTTTCGGGGAAATGTGCGCGGAACCCCTATTTGTTTATTTTTCTAAATACATTCAAATATGTATCCGCTCATGAGACAATAACCCTGATAAATGCTTCAATAATATTGAAAAAGGAAGAGTATGAGTATTCAACATTTCCGTGTCGCCCTTATTCCCTTTTTTGCGGCATTTTGCCTTCCTGTTTTTGCTCACCCAGAAACGCTGGTGAAAGTAAAAGATGCTGAAGATCAGTTGGGTGCACGAGTGGGTTACATCGAACTGGATCTCAACAGCGGTAAGATCCTTGAGAGTTTTCGCCCCGAAGAACGTTTTCCAATGATGAGCACTTTTAAAGTTCTGCTATGTGGCGCGGTATTATCCCGTGTTGACGCCGGGCAAGAGCAACTCGGTCGCCGCATACACTATTCTCAGAATGACTTGGTTGAGTACTCACCAGTCACAGAAAAGCATCTTACGGATGGCATGACAGTAAGAGAATTATGCAGTGCTGCCATAACCATGAGTGATAACACTGCGGCCAACTTACTTCTGACAACGATCGGAGGACCGAAGGAGCTAACCGCTTTTTTGCACAACATGGGGGATCATGTAACTCGCCTTGATCGTTGGGAACCGGAGCTGAATGAAGCCATACCAAACGACGAGCGTGACACCACGATGCCTGtAGCAATGGCAACAACGTTGCGCAAACTATTAACTGGCGAACTACTTACTCTAGCTTCCCGGCAACAATTAATAGACTGGATGGAGGCGGATAAAGTTGCAGGACCACTTCTGCGCTCGGCCCTTCCGGCTGGCTGGTTTATTGCTGATAAATCTGGAGCCGGTGAGCGTGGGTCTCGCGGTATCATTGCAGCACTGGGGCCAGATGGTAAGCCCTCCCGTATCGTAGTTATCTACACGACGGGGAGTCAGGCAACTATGGATGAACGAAATAGACAGATCGCTGAGATAGGTGCCTCACTGATTAAGCATTGGTAACTGTCAGACCAAGTTTACTCATATATACTTTAGATTGATTTAAAACTTCATTTTTAATTTAAAAGGATCTAGGTGAAGATCCTTTTTGATAATCTCATGACCAAAATCCCTTAACGTGAGTTTTCGTTCCACTGAGCGTCAGACCCCGTAGAAAAGATCAAAGGATCTTCTTGAGATCCTTTTTTTCTGCGCGTAATCTGCTGCTTGCAAACAAAAAAACCACCGCTACCAGCGGTGGTTTGTTTGCCGGATCAAGAGCTACCAACTCTTTTTCCGAAGGTAACTGGCTTCAGCAGAGCGCAGATACCAAATACTGTCCTTCTAGTGTAGCCGTAGTTAGGCCACCACTTCAAGAACTCTGTAGCACCGCCTACATACCTCGCTCTGCTAATCCTGTTACCAGTGGCTGCTGCCAGTGGCGATAAGTCGTGTCTTACCGGGTTGGACTCAAGACGATAGTTACCGGATAAGGCGCAGCGGTCGGGCTGAACGGGGGGTTCGTGCACACAGCCCAGCTTGGAGCGAACGACCTACACCGAACTGAGATACCTACAGCGTGAGCATTGAGAAAGCGCCACGCTTCCCGAAGGGAGAAAGGCGGACAGGTATCCGGTAAGCGGCAGGGTCGGAACAGGAGAGCGCACGAGGGAGCTTCCAGGGGGAAACGCCTGGTATCTTTATAGTCCTGTCGGGTTTCGCCACCTCTGACTTGAGCGTCGATTTTTGTGATGCTCGTCAGGGGGGCGGAGCCTATGGAAAAACGCCAGCAACGCGAGCTCgtatggacatattgtcgttagaacgcggctacaattaatacataaccttatgtatcatacacatacgatttaggggacactatag

**Supplementary Table 2. Primers used for constructing mouse mitoRCA-seq library.**

| Pr_id | Pr_f pos | Pr_f seq | Pr_r pos | pr_r seq |
| --- | --- | --- | --- | --- |
| Pr01 | 17-37 | AACAAAGCAAAGCACTGAAAA | 880-900 | GCGTACTTCATTGCTCAATTC |
| Pr02 | 691-711 | CTTCAGCAAACCCTAAAAAGG | 1634-1654 | TTTTATGTTGAGCTTGAACGC |
| Pr03 | 1752-1772 | ATTCCAATTCTCCAGGCATAC | 2553-2573 | CCTTTCGTACTGGGAGAAATC |
| Pr04 | 2347-2367 | TTTGATCAACGGACCAAGTTA | 3404-3424 | ATAAAGAATAACGCGAATGGG |
| Pr05 | 3134-3154 | ACTATTCGGAGCTTTACGAGC | 4112-4132 | GGCCAGGAGGATAATTATTGA |
| Pr06 | 4068-4088 | CAACTGAAGCAGCAACAAAAT | 4908-4928 | GGGGTAGGGTTATTGTGCTTA |
| Pr07 | 4908-4928 | TAAGCACAATAACCCTACCCC | 5699-5719 | GGTGGGTAGACTGTTCATCCT |
| Pr08 | 5555-5575 | AGGCTTTGGAAACTGACTTGT | 6400-6420 | TGTCAAGGGATGAGTTGGATA |
| Pr09 | 6400-6420 | TATCCAACTCATCCCTTGACA | 7400-7420 | TAGCAGTCGTAGTTCACCAGG |
| Pr10 | 7309-7329 | CATAGGGCACCAATGATACTG | 8237-8257 | CAGCTCATAGTGGAATGGCTA |
| Pr11 | 8012-8032 | TCCTATTCCCATCCTCAAAAC | 8913-8933 | TGTTGGTACGAGGCTAGAATG |
| Pr12 | 9074-9094 | AAACCACATAAATCAAGCCCT | 9943-9963 | TGTGGATATTAGGTGAGAGCG |
| Pr13 | 9587-9607 | TACAAGCTCTGCACGTCTACC | 10714-10734 | ATGAAGCGTCTAAGGTGTGTG |
| Pr14 | 10643-10663 | ACTGCTAATTGCCCTCATCTT | 11436-11456 | GTGTGAGGGTTGGAGGTTAAT |
| Pr15 | 11208-11228 | CTTCAAATGGTCTTCCCACTT | 12065-12085 | TTGATGTTTGGGTCTGAGTGT |
| Pr16 | 12156-12176 | CAACTTTTCATTGGCTGAGAA | 12955-12975 | TAATTAGTAGGGCTCAGGCGT |
| Pr17 | 13673-13693 | ACTCCAACATCATCAACCTCA | 14650-14670 | TCAAGGTGGCTTTGTCTACTG |
| Pr18 | 14603-14623 | CCCATATATTGGAACAACCCT | 15479-15499 | AGCTTATATGCTTGGGGAAAA |
| Pr19 | 15358-15378 | AAGAAGAAGGAGCTACTCCCC | 16245-16265 | GAGTTTTGGTTCACGGAACAT |

Note: _f and _r refer to the forward and reverse primer respectively. pos denotes position of the primer in the mtDNA.

**Supplementary Table 3. Primers used for constructing fruit fly mitoRCA-seq library.**

| Pr_id | Pr_f pos | Pr_f seq | Pr_r pos | Pr_r seq |
| --- | --- | --- | --- | --- |
| Pr01 | 7-28 | TTGCCTGATAAAAAGGATTACC | 1103-1126 | GCGGAATAACAAATTCGTAAATAA |
| Pr02 | 978-1000 | TTAGGAGGATTACCTCCATTTTT | 2056-2079 | TCCTGCTAGTACTGGAAGTGATAA |
| Pr03 | 1830-1850 | AGCTGGAACAGGATGAACTGT | 2946-2966 | GGCGGAGTATTTTGGTATCAT |
| Pr04 | 2957-2977 | ATACTCCGCCAGCTGAACATA | 4080-4100 | AGCTAAGGGGTCGAATACAGA |
| Pr05 | 3705-3727 | CGATTGTAATTGAAAGTGTTCCT | 4786-4806 | CCGATAGCTCCTGTTAATGGT |
| Pr06 | 4913-4933 | CGAGATGTATCACGAGAAGGA | 5999-6020 | TTTTGAATGCAAATCAAATGTT |
| Pr07 | 5736-5756 | TGATCCAAAATCTTCATCTCG | 6901-6921 | GGTGATTTAAATTGCGGTAGA |
| Pr08 | 7690-7710 | CCCAATTCGATTAGATAACGC | 8896-8916 | AAGCTCCAGTTTCTGGGTCTA |
| Pr09 | 8703-8723 | TGAGCAACAGATGAATAAGCA | 9917-9938 | TTGGGGATTAATGAAAAAGAAA |
| Pr10 | 9618-9638 | AGGCCCCTTCACATACTCTAA | 10738-10758 | ACCGTTAGCATGTAAAGTTCG |
| Pr11 | 10581-10602 | TTCAAGATGATGAAATTTTGGA | 11828-11848 | CGAGGAACTTTACCTCGATTT |
| Pr12 | 11519-11539 | CTCGACCAGTTGAAGAACCTT | 12770-12790 | CGAAAGGACCAAATATCAAAA |
| Pr13 | 14440-14460 | AAGTAAGGTCCATCGTGGATT | 15700-15723 | AAATTTATGAATAGGGGGAATAAA |

Note: _f and _r refer to the forward and reverse primer respectively. pos denotes position of the primer in the mtDNA.

**Supplementary Table 4. Counts of sites with background error frequency in the control samples.**

| **Control Sample** | **SNV frequency* > 0.3%** | **0.3% ≤ SNV frequency* < 1%** | **1% ≤ SNV frequency*  ≤ 10%** | **SNV frequency* ≥ 10%** | **False positive sites** |
| --- | --- | --- | --- | --- | --- |
| **Plasmid** | 14 | 0 | 1 | 13 (all close to 100%) | 1 |
| **PhiX_01** | 7 | 2 | 2 | 3 (close to 100%) | 4 |
| **PhiX_02** | 4 | 1 | 0 | 3 (close to 100%) | 1 |

Note: The pTEsindbisGFP plasmid is 14,571 bp in length. The full-length PhiX genome consists of 5,386 bp. PhiX_01 and PhiX_02 represent two independent sequencing runs. False positive sites shown in the last column is calculated by subtracting the number in the 5^th^ column from the number in the 2^nd^ column. If the frequency of a mutation is close to 100%, it is unlikely to be a real mutation but mis-annotation of the reference sequence. Therefore, these sites (the 5^th^ column) are removed to determine the true mutation site(s).

* Additional criteria were applied: (1) the nucleotide supporting the mutation should have a sequencing and mapping score greater than 30; (2) the mutation site should also be supported by three or more BURs, and (3) the mutation frequency supported by best-unique reads should be > 0.2% to avoid sporadic sequencing errors due to higher coverage (see Methods for details).

**Supplementary Table 5. Summary of data analysis results based on Figure 1b.**

| **Sample ID*** | **Platform** | **Raw reads** | **Filtrated reads (≥Q30)**** | **Reads mapped to mtDNA** | **% of read mapped to mtDNA** | **The candidate point mutation sites***** | **The candidate point mutation events****** |
| --- | --- | --- | --- | --- | --- | --- | --- |
| Wild type liver  Bio-rep #1  PCR library | HiSeq | 46,114,144 | 40,605,237 | 29,001,626 | 71.4% | 450 | 459 |
| Wild type liver  Bio-rep #2  PCR library | HiSeq | 54,2367,74 | 48,047,091 | 31,035,904 | 64.6% | 433 | 435 |
| Wild type brain  Bio-rep #1  PCR library | HiSeq | 46,660,750 | 40,802,510 | 24,292,549 | 59.5% | 354 | 363 |
| Wild type brain  Bio-rep #2  PCR library | HiSeq | 52,282,264 | 45,606,999 | 30,813,400 | 67.6% | 322 | 331 |
| Polg mutant liver  Bio-rep #1  PCR library | HiSeq | 53,510,354 | 46,982,949 | 25,779,653 | 54.9% | 1079 | 1090 |
| Polg mutant liver  Bio-rep #2  PCR library | HiSeq | 62,559,038 | 54,901,572 | 32,874,140 | 59.9% | 1222 | 1240 |
| Polg mutant brain  Bio-rep #1  PCR library | HiSeq | 46,002,518 | 40,572,318 | 27,660,620 | 68.2% | 786 | 799 |
| Polg mutant brain  Bio-rep #2  PCR library | HiSeq | 42,477,114 | 37,186,819 | 25,189,598 | 67.7% | 766 | 779 |
| Wild type liver  Bio-rep #1  PCR-free library | MiSeq | 2,144,392 | 2,074,164 | 1,991,043 | 96.0% | 366 | 368 |
| Wild type liver  Bio-rep #2  PCR-free library | MiSeq | 1,199,802 | 1,162,292 | 1,077,872 | 92.7% | 460 | 460 |
| Wild type brain  Bio-rep #1  PCR-free library | MiSeq | 2,931,508 | 2,830,906 | 2,495,261 | 88.1% | 208 | 211 |
| Wild type brain  Bio-rep #2  PCR-free library | MiSeq | 2,205,930 | 2,126,039 | 1,800,164 | 84.7% | 215 | 216 |
| Polg mutant liver  Bio-rep #1  PCR-free library | MiSeq | 1,423,680 | 1,358,236 | 397,738 | 29.3% | 1,318 | 1335 |
| Polg mutant liver  Bio-rep #2  PCR-free library | MiSeq | 1,837,062 | 1,752,159 | 668,039 | 38.1% | 1,381 | 1397 |
| Polg mutant brain  Bio-rep #1  PCR-free library | MiSeq | 2,349,324 | 2,254,029 | 2,089,226 | 92.7% | 516 | 520 |
| Polg mutant brain  Bio-rep #2  PCR-free library | MiSeq | 2,202,388 | 2,122,556 | 2,039,577 | 96.1% | 552 | 560 |
| **Total** |  | **420,137,042** | **370,385,876** | **239,206,410** | **64.6%** |  |  |

*mitoRCA-seq libraries derived from mice, Bio-rep #1 and #2 denote biological replicate 1 and 2, respectively.

**Reads that have average quality score **≥** 30.

***The sites of mouse mtDNA that have candidate point mutation. One site can have more than one mutation events.

****The point mutation events in each sample, one site can have multiple point mutation events. Only a few sites have more than two point mutation events.

**Supplementary Table 6. Mapping summary of data from 1 ng, 5 ng and 50 ng of mouse liver total DNA.**

| **Sample*** | **Filtrated reads (≥Q30)**** | **Reads mapped to mtDNA** | **% of read mapped to mtDNA** |
| --- | --- | --- | --- |
| M_Liver_1ng_R1 | 4,021,126 | 2,773,525 | 68.97% |
| M_Liver_1ng_R2 | 3,762,246 | 2,604,458 | 69.23% |
| M_Liver_5ng_R1 | 4,186,212 | 3,944,017 | 94.21% |
| M_Liver_5ng_R2 | 4,036,401 | 3,734,066 | 92.51% |
| M_Liver_50ng_R1 | 3,227,236 | 3,106,477 | 96.26% |
| M_Liver_50ng_R2 | 3,061,212 | 2,931,953 | 95.78% |

*Wild-type mouse liver total DNA was used for library construction. Libraries were sequenced by Illumina MiSeq. R1, R2 denotes read 1 and read 2, respectively.

**Supplementary Table 7. Mutational load in D-loop region compared with other regions**.

|  | **Fold (Mut/WT)** |
| --- | --- |
| **Coding Brain** | 1.6076 |
| **Coding Liver** | 1.8296 |
| **D-loop Brain** | 1.3742 |
| **D-loop Liver** | 1.3967 |
| **tRNA Brain** | 1.6190 |
| **tRNA Liver** | 1.5238 |
| **rRNA Brain** | 1.4208 |
| **rRNA Liver** | 1.3601 |

Note: Mutation sites with mutation frequency smaller than 10% were used for mutational load calculation since sites with higher mutation frequency were unlikely caused by mutated *Polg* gene.

**Supplementary Table 8. Comparison of reads contamination from Numts.**

| **Library type** | **Sample*** | **Total reads** | **Quality reads** | **Map to mtDNA** | **Numts origin** |
| --- | --- | --- | --- | --- | --- |
| **Capture-based**** | ERR086292 | 787,884 | 786,896 | 362,782 | 4,728 (0.60%) |
|  | ERR086300 | 765,705 | 764,846 | 218,513 | 7,735 (1.01%) |
|  | ERR086304 | 806,673 | 805,484 | 306,398 | 9,185 (1.14%) |
| **Long-range PCR**** | ERR086280 | 829,917 | 818,159 | 702,038 | 4,948 (0.60%) |
|  | ERR086285 | 714,478 | 704,752 | 593,092 | 3,662 (0.52%) |
|  | ERR086287 | 791,034 | 779,766 | 690,096 | 3,106 (0.40%) |
| **mitoRCA-seq**** | Brain_mut1 | 46,002,518 | 44,092,891 | 39,208,772 | 137,142 (0.31%) |
|  | Brain_mut2 | 42,477,114 | 40,567,659 | 35,857,785 | 107,832 (0.27%) |
|  | Brain_WT1 | 46,660,750 | 44,535,805 | 34,639,043 | 128,823 (0.29%) |
|  | Brain_WT2 | 52,282,264 | 49,805,959 | 43,949,652 | 162,378 (0.33%) |
|  | Liver_mut1 | 53,510,354 | 51,229,112 | 36,887,041 | 98,130 (0.19%) |
|  | Liver_mut2 | 62,559,038 | 59,791,461 | 46,818,279 | 122,856 (0.21%) |
|  | Liver_WT1 | 46,114,144 | 44,134,528 | 41,192,198 | 119,201 (0.27%) |
|  | Liver_WT2 | 54,2367,74 | 52,113,763 | 44,125,373 | 137,536 (0.26%) |

*Access number of publically available datasets from Li et al.^1^

**Since majority reads in Capture-based and Long-range PCR based datasets have quality score just above 20, we thus used average quality score >20 as a filter threshold for all three library types.

**Supplementary Table 9. The relative abundance of C**→**T transitions in individual samples.**

|  | **Total # of mutation sites** | **C→T** | **C→T%** | **T→C** | **T→C%** |
| --- | --- | --- | --- | --- | --- |
| **BrMut1_spec** | 596 | 259 | 43.46 | 60 | 10.07 |
| **BrMut2_spec** | 571 | 242 | 42.38 | 82 | 14.36 |
| **BrWT1_spec** | 164 | 14 | 8.54 | 57 | 34.76 |
| **BrWT2_spec** | 127 | 13 | 10.24 | 46 | 36.22 |
| **LvMut1_spec** | 835 | 414 | 49.58 | 69 | 8.26 |
| **LvMut2_spec** | 918 | 467 | 50.87 | 93 | 10.13 |
| **LvWT1_spec** | 206 | 17 | 8.25 | 70 | 33.98 |
| **LvWT2_spec** | 129 | 14 | 10.85 | 52 | 40.31 |

*These data are based on low-cycle PCR results.

**Supplementary Table 10. Definition of amino acid’s property.**

| **Amino Acid** | **Property** |
| --- | --- |
| Glycine | Hydrophilic |
| Alanine | Hydrophobic |
| Valine | Hydrophobic |
| Leucine | Hydrophobic |
| Isoleucine | Hydrophobic |
| Phenylalanine | Hydrophobic |
| Tryptophan | Hydrophobic |
| Tyrosine | Hydrophobic |
| Aspartic acid | Acidic |
| Histidine | Basic |
| Asparagine | Hydrophilic |
| Glutamic acid | Acidic |
| Lysine | Basic |
| Glutamine | Hydrophilic |
| Methionine | Hydrophobic |
| Arginine | Basic |
| Serine | Hydrophilic |
| Threonine | Hydrophilic |
| Cysteine | Hydrophilic |
| Proline | Hydrophobic |
| Terminator(*) | nonsense |

**Supplementary Table 11. *Polg* mutant mice have higher level of small InDels than wild-type mice.**

| Samples | InDel Cut-off frequency | # of InDels | # of 1nt InDels | # of 2nt InDels | #of 3nt InDels |
| --- | --- | --- | --- | --- | --- |
| Brmut1 | 0.1% | 250 | 237 | 10 | 3 |
| Brmut2 | 0.1% | 252 | 237 | 12 | 3 |
| BrWT1 | 0.1% | 120 | 113 | 7 | 0 |
| BrWT2 | 0.1% | 131 | 120 | 10 | 1 |
| Lvmut1 | 0.1% | 258 | 246 | 9 | 3 |
| Lvmut2 | 0.1% | 305 | 285 | 17 | 3 |
| LvWT1 | 0.1% | 161 | 148 | 10 | 3 |
| LvWT2 | 0.1% | 138 | 126 | 9 | 3 |
|  |  |  |  |  |  |
| Brmut1 | 0.2% | 109 | 105 | 4 | 0 |
| Brmut2 | 0.2% | 110 | 101 | 7 | 2 |
| BrWT1 | 0.2% | 67 | 63 | 4 | 0 |
| BrWT2 | 0.2% | 67 | 63 | 4 | 0 |
| Lvmut1 | 0.2% | 118 | 112 | 4 | 2 |
| Lvmut2 | 0.2% | 132 | 122 | 8 | 2 |
| LvWT1 | 0.2% | 76 | 70 | 6 | 0 |
| LvWT2 | 0.2% | 69 | 65 | 4 | 0 |
|  |  |  |  |  |  |
| Brmut1 | 0.3% | 66 | 62 | 4 | 0 |
| Brmut2 | 0.3% | 69 | 62 | 5 | 2 |
| BrWT1 | 0.3% | 46 | 43 | 3 | 0 |
| BrWT2 | 0.3% | 46 | 42 | 4 | 0 |
| Lvmut1 | 0.3% | 74 | 68 | 4 | 2 |
| Lvmut2 | 0.3% | 74 | 68 | 4 | 2 |
| LvWT1 | 0.3% | 51 | 48 | 3 | 0 |
| LvWT2 | 0.3% | 45 | 41 | 4 | 0 |

Note: Three different frequency cut-offs (0.1%, 0.2% and 0.3%) of small InDels were applied to evaluate the changes between wild-type and *Polg* mutant mice. Br and Lv is abbreviation of brain and liver, respectively. Four samples are shown for each tissue, consisting of biological replicates derived from two wild-type (WT1 and WT2) and two *Polg* mutant mice (mut1 and mut2). For each cut-off, *Polg* mutant mice have higher number (#) of small InDels than wild-type, both in brain and liver.

**Supplementary Methods**

**Total DNA extraction from mouse and human tissues**

Brain and live tissues were taken from wild type and *Polg* mutant mice of 6 weeks old. QIAamp DNA Blood Mini kit (Qiagen) was used to extract total DNA from mouse and human tissues according to the manufacturer’s protocol. Briefly, 200 μl AL buffer was added to liver or brain sample (~20 mg) and the tissue were homogenized with one steel bead in a 2 ml tube at Qiagen TissueLyser for 2min at 50 oscillations per second. After column purification, total genomic DNA was eluted with 200 μl water and the DNA concentration is quantified by Nanodrop spectrophotometer (Thermo Fisher). 100 ng, 50 ng, 5 ng and 1 ng of total gDNA was used for mitoRCA-seq library construction.

**Total DNA extraction from Drosophila**

Three adult flies were homogenized in 100 μl lyses buffer (0.1 M Tris-HCl, pH 9.0; 0.1 M EDTA; 1% SDS) and incubated at 65°C for 30 minutes after homogenization. 14 μl of 8 M potassium acetate were added to homogenate and left on ice for 30 minutes. After 15 minutes centrifugation at 4°C, the supernatant was transferred into a fresh tube, and DNA was precipitated by adding 50 μl isopropanol and spinning for 5 minutes at room temperature. After washing with 70% ethanol, the pellet was dried and resuspended in 50μl nuclease-free water. Total DNA concentration was quantified by Nanodrop Spectrophotometers (Thermo Fisher).

**RCA with mitochondrial-specific oligos**

For constructing mitoRCA-seq library, 100 ng of total DNA was used as template for RCA amplification. Briefly, a 50 μl reaction mix containing 100 ng total DNA, 1x Phi29 buffer (NEB), 0.2 μg/ml BSA, 1 mM dNTP (NEB) and 25 μM specific oligos (20 primer pairs for the pTEsindbisGFP plasmid, 19 primer pairs for mouse mtDNA and 13 primer pairs for *Drosophila* mtDNA) was denatured for 3 min at 95°C. After cooling down at room temperature for 10 minutes, 1 μl of Phi29 DNA polymerase (10 unit/μl, NEB) was added to the reaction mix, which was then incubated at 37°C for 16 hours followed by 65°C for 10 minutes to heat inactivate the enzyme. For the titration test in Supplementary Figure 2, the input DNA amounts were titrated from 100ng to 0.1pg. Note: The REPLI-g Mitochondrial DNA kit (Qiagen), which is based on RCA amplification, can also be used to selectively enrich human and mouse mtDNAs from total genomic DNA.

**Restriction enzyme digestions**

Different restriction enzymes were selected to digest the RCA products according to the species’ mtDNA reference sequence to generate two distinguishable bands in 0.5% agarose gel. For mouse, EcoRV (NEB) was used to digest RCA products into two long fragments (9.5kb and 6.8kb). For fruit fly, NdeI (NEB) and EcoRV (NEB) were used to digest RCA products into two long fragments (10kb and 9.8kb). All digestion reactions were carried out according to the manufacturer’s protocol.

**MitoRCA-seq library construction**

Size selection of mitochondrial specific DNA fragments was carried out with 0.5% agarose gel. Zymoclean large fragment gel purification kit (ZYMO research) was used to purify the excised bands. The resulting DNA fragments were then sheared by Covaris S2 instrument into small fragments with a size peaked at 300 bp. End-IT kit from Epicentre was applied to perform end repairing, followed by size selection for 200-400bp in a 2% agarose gel. We did A-tailing for the blunt-ended DNA by Klenow exo- DNA polymerase (Epientre). Illumina index adaptors were then ligated, followed by 8-12 cycle PCR with Phusion High Fidelity DNA polymerase (NEB). Size selection (300-500bp) of the final PCR products was performed in a 2% agarose gel to prepare the mitoRCA-seq libraries, which were subjected to deep sequencing by Illumina HiSeq2000 or MiSeq instrument. For PCR-free libraries, we followed the library construction protocol of NuGEN Encore Rapid Library Systems after the fragmentation step.

**mitoRCA-seq starting with limited DNA**

To test what’s the minimum amount of total DNA can be used for mitoRCA-seq library construction, we setup RCA reaction similar to library construction except that we started with 100 ng, 1 ng, 10 pg or 0.1 pg total DNA. We tested this with *Drosophila* total DNA and used EcoRI (NEB) for digestion since it will generate four fragments of different sizes to better visualize digestion pattern.

**Data analysis pipeline**

The raw reads were first filtered based on their average quality score, and the sequence reads with an average quality score greater than 30 were kept for further analysis. The quality-filtered reads were aligned to the reference mitochondrial genome (UCSC, mm10) by BWA using default parameters^2^, and the uniquely mapped reads were used for further analysis. The mpileup package of SAMtools^3^ was used for the sequence variation discovery with the default parameters except with the *–d 200,000* to accommodate the actual sequencing depth. To remove the artificial variants caused by the sequencing errors, the mapping quality score at a point mutation site should be greater than 30. To remove PCR artifacts which tend to generate the same fragments and lead to duplicate reads in the final step, we only kept one read of the best average quality (best-unique reads) for those duplicate reads passing the 30-average-quality-score filtration. And the best-unique reads also go through the same analysis pipeline used by the total reads. Taken together, the potential point mutation sites should follow the criteria below: i) the nucleotide supporting the mutation should have a sequencing and mapping score greater than 30; ii) have a mutation frequency greater than 0.3%; and iii) the mutation site should also be supported by both ≥ 3 best-unique reads and mutation frequency supported by best-unique reads should be > 0.2% to avoid random sequencing error due to higher coverage.

**Calculation of Numts contamination in mouse and human nuclear genome**

To evaluate the prevalence of Numts in the nuclear genome, the whole mitochondrial genome (mm10 chrM download from UCSC genome browser for mouse, and human mtDNA (NC_012920.1) download from NCBI) was aligned to the nuclear genome (mm10 for mouse and hg19 for human) by NCBI Blast-2.2.27+ program using the default parameters. The aligned fragments smaller than 50bp were filtered out (only 1 such fragment). For mouse, a total of 43 nuclear mitochondrial like fragments (Numts) were found, which collectively is equivalent to 1.89 x of the mitochondrial genome in size, covering 84.5% of the mitochondrial genome. As for human, 169 Numts were found in human genome with the same criteria, which are 15.4x as the human mitochondrial genome in size, covering 99.99% of the human mitochondrial genome.

**Calculate overlapping between Numts and mtDNA**

To find out the potential nuclear mitochondrial like fragments (Numts) in the genome, the mouse mitochondrial genome (mm10 chrM) was aligned to the mouse nuclear genome (mm10) by BLASTn (NCBI Blast-2.2.26) program using the default parameters. All hits with length over 50bp were kept as Numt fragments, a total of 143 Numts were defined in this way, with about 2.5 x of the mitochondrial genome in size. *Diff-seq* package of *EMBOSS* suite of 6.5.7 version was used to identify single nucleotide variations between mitochondrial genome and the candidate Numts point mutations. Since the datasets from Li et al.^1^ are human origin, we used human mtDNA (NC_012920.1) and human genome (hg19) for the alignment. To evaluate the possible contamination of reads from Numts, we aligned the Q20-filtered reads to Numts with BWA 0.6.1^2^. Reads with perfect match to Numts were extracted out and aligned to the mitochondrial genome. After filtering out reads that perfect match to the mitochondrial genome, the remaining reads were considered as contaminations from Numts.

**References:**

1. Li, M., Schroeder, R., Ko, A. & Stoneking, M. Fidelity of capture-enrichment for mtDNA genome sequencing: influence of Numts. *Nucleic Acids Res.* **40**, e137 (2012).

2. Li, H. & Durbin, R. Fast and accurate short read alignment with Burrows-Wheeler transform. *Bioinformatics* **25**, 1754-1760 (2009).

3. Li, H. *et al.* The Sequence Alignment/Map format and SAMtools. *Bioinformatics* **25**, 2078-2079 (2009).
